# Toward Overcoming Treatment Failure in Rheumatoid Arthritis

**DOI:** 10.3389/fimmu.2021.755844

**Published:** 2021-12-23

**Authors:** Zhuqian Wang, Jie Huang, Duoli Xie, Dongyi He, Aiping Lu, Chao Liang

**Affiliations:** ^1^ Department of Biology, School of Life Sciences, Southern University of Science and Technology, Shenzhen, China; ^2^ Institute of Integrated Bioinfomedicine and Translational Science (IBTS), School of Chinese Medicine, Hong Kong Baptist University, Hong Kong, Hong Kong SAR, China; ^3^ Law Sau Fai Institute for Advancing Translational Medicine in Bone and Joint Diseases, School of Chinese Medicine, Hong Kong Baptist University, Hong Kong, Hong Kong SAR, China; ^4^ Institute of Arthritis Research in Integrative Medicine, Shanghai Academy of Traditional Chinese Medicine, Shanghai, China; ^5^ Department of Rheumatology, Shanghai Guanghua Hospital of Integrative Medicine, Shanghai, China; ^6^ Guangdong-Hong Kong-Macau Joint Lab on Chinese Medicine and Immune Disease Research, Guangzhou, China

**Keywords:** rheumatoid arthritis, DMARDs, biomarker, treatment failure, precision medicine

## Abstract

Rheumatoid arthritis (RA) is an autoimmune disorder characterized by inflammation and bone erosion. The exact mechanism of RA is still unknown, but various immune cytokines, signaling pathways and effector cells are involved. Disease-modifying antirheumatic drugs (DMARDs) are commonly used in RA treatment and classified into different categories. Nevertheless, RA treatment is based on a “trial-and-error” approach, and a substantial proportion of patients show failed therapy for each DMARD. Over the past decades, great efforts have been made to overcome treatment failure, including identification of biomarkers, exploration of the reasons for loss of efficacy, development of sequential or combinational DMARDs strategies and approval of new DMARDs. Here, we summarize these efforts, which would provide valuable insights for accurate RA clinical medication. While gratifying, researchers realize that these efforts are still far from enough to recommend specific DMARDs for individual patients. Precision medicine is an emerging medical model that proposes a highly individualized and tailored approach for disease management. In this review, we also discuss the potential of precision medicine for overcoming RA treatment failure, with the introduction of various cutting-edge technologies and big data.

## Introduction

Rheumatoid arthritis (RA), an autoimmune disorder that preferentially attacks the joints, affects approximately 1% of people worldwide (1). RA patients experience morning stiffness in the early stage, which manifests as facet joint pain, swelling, and synovitis. In the late stage, small focal necrosis and granulation tissue pannus formation appear, spreading to the cartilage surface, accompanied by symmetrical polyarticular swelling, bone erosion and pain mainly in the interphalangeal and metacarpophalangeal joints and limited mobility (2). Finally, granulation tissue and fibrous tissue adhesion appear on the articular surface, forming deformity symptoms such as ankylosis and joint subluxation. Most patients also present with extra-articular multisystem involvement in skin, blood, kidneys and lungs, further aggravating the condition (2). 

The exact mechanism of RA development is unknown, but both genetic and environmental factors are contributory. Various proinflammatory cytokines and immune cells are involved in RA pathophysiology (3). In the progress of RA, the synovium is infiltrated by leukocytes and synovial fluid is inundated with pro-inflammatory cytokines, such as tumor necrosis factor (TNF), interleukin (IL)-6, IL-17 and IL-1β (4). These cytokines induce an inflammatory cascade characterized by interactions of fibroblast-like synoviocytes (FLS) with innate immune cells, including macrophages, monocytes, dendritic cells and mast cells, as well as adaptive immune cells such as B and T cells. TNF also promotes bone resorption and bone erosion. IL-1 indirectly stimulates osteoclast formation. IL-6 aggravates pathogenic effects in RA by enhancing the inflammatory effects of IL-1 and TNF (4). An imbalance between osteoblasts/osteoclasts and regulatory T (Treg)/T helper (Th)17 cells are typical characteristics of RA (5).

RA requires the combined effects of different signaling pathways, such as receptor activator of nuclear factor kappa B ligand (RANKL)/receptor activator of nuclear factor kappa B (RANK)/osteoprotegerin (OPG) and IL-6/glycoprotein 130 (gp130)/janus kinase (JAK)/signal transducer and activator of transcription (STAT). For RANKL/RANK/OPG signaling, binding of RANKL to RANK induces nuclear factor kappa B (NF-κB) activation, which upregulates levels of pro-inflammatory cytokines (IL-1, IL-6 and TNF) and mediates proliferation of T and B cells (6). OPG binds explicitly to RANKL and inhibits RANKL activity by preventing its binding to RANK. RANKL promotes the differentiation and production of osteoclasts. For IL-6/gp130/JAK/STAT signaling, IL-6 binds to IL-6 receptor (IL-6R). The IL-6/IL-6R complex interacts with gp130 to induce its dimerization and initiate intracellular signaling *via* JAK/STAT pathway, thus increasing T cell activity, inhibiting FLS apoptosis, allowing B cell maturation and stimulating differentiation of naive T cells into Th17 cells (7).

To assess disease activity of RA, the American College of Rheumatology (ACR), the European League Against Rheumatism (EULAR) and the World Health Organization/International League Against Rheumatism (WHO/ILAR) have established a core set of variables, which include swollen joint count (SJC), tender joint count (TJC), physician’s global assessment of disease activity (PhGA), patient’s global assessment of disease activity (PtGA), patient’s assessment of pain, patient’s assessment of physical function, and level of an acute phase reactant (APR, either C-reactive protein (CRP) or erythrocyte sedimentation rate (ESR)) (8). Based on these variables, composite measurement tools with strong clinimetric properties have been developed. Among these tools are dichotomous indices like the ACR response criteria (ACR20, 50 and 70) (9), and continuous scores like the Disease Activity Score for 28 joints (DAS28), the Clinical Disease Activity Index (CDAI) and the Simplified Disease Activity Index (SDAI) (10). ACR20, 50 and 70 are based on improvement of at least 20%, 50% and 70% in both TJC and SJC, and three of the five additional core set of variables listed above, respectively. DAS28 considers TJC and SJC of 28 joints, PtGA, plus level of an APR (either ESR or CRP) (11). CDAI is based on the simple summation of TJC and SJC of 28 joints, along with PhGA and PtGA (12). SDAI is the arithmetic sum of TJC and SJC of 28 joints, PhGA, PtGA and level of an APR (CRP) (12). These tools allow better standardization and interpretation of disease activity of RA and patient response to therapy.

The EULAR has updated its recommendations for the management of RA in 2019, which are regarded as the main guidelines worldwide. In this update, most of the recommendations remain unchanged when reviewing its first version one decade ago and the updates in 2013 and 2016. The target of treatment remains as sustained remission (according to the ACR-EULAR definition) or low disease activity, and the major focus continues to be pharmacological therapy with disease-modifying antirheumatic drugs (DMARDs) (13). The DMARDs are divided into conventional synthetic (cs) DMARDs (such as methotrexate, leflunomide and sulfasalazine), biological (b) DMARDs [TNF inhibitors (infliximab, etanercept, adalimumab, certolizumab pegol and golimumab), a T cell co-stimulation inhibitor (abatacept), a cluster of differentiation 20 (CD20) inhibitor (rituximab), IL-6R inhibitors (tocilizumab and sarilumab) and biosimilar (bs) DMARDs)] and targeted synthetic (ts) DMARDs [JAK inhibitors (such as tofacitinib, baricitinib and upadacitinib)] (13).

Over the past years, the management of RA has progressed remarkably, encompassing the development of the above measurement tools and approvals of various DMARDs. However, the response is not universal for any treatment option. A large number of clinical trials have demonstrated that substantial proportions of RA patients experience treatment failure after receiving csDMARDs and even bDMARDs and tsDMARDs (14–19). Treatment failure is defined as nonresponse or limited efficacy (16, 17, 20), including initial lack of response, responsiveness over time, and inadequate response (partial response). To date, continuous efforts have been made toward overcoming treatment failure in RA patients, such as identification of biomarkers for response or nonresponse to DMARDs, exploration of the reasons for loss of efficacy, development of sequential or combinational DMARDs strategies either within the same or different mechanistic class, and approval of new DMARDs (13). Some of them provide valuable insights that can help to improve the design of future clinical trials and enable accurate clinical medication (21).

Due to the striking heterogeneity of RA, people realize that the current efforts are far from enough to recommend specific DMARDs for individual patients, which is also highlighted by EULAR as an important issue to be addressed in the future (13). Precision medicine, also called personalized medicine, is an emerging medical model that proposes a highly individualized and tailored approach for patient management by accounting for individual variability in genes, environment, and lifestyle, instead of a one‐drug‐fits‐all model (22). It involves the ability to classify individuals into subpopulations that are susceptible or responsive to a specific treatment (23). Precision medicine is in its infancy and has not become a routine practice in RA. But it is anticipated that precision medicine would have tremendous potential to address the treatment failure for RA (24).

In this review, we summarize the current efforts in identifying biomarkers for DMARDs, exploring the reasons for loss of efficacy, developing sequential or combinational DMARDs strategies and approving of new DMARDs, toward overcoming treatment failure in RA. We also discuss the opportunities and advantages of precision medicine approaches to make a breakthrough in diagnosis, prognosis, and treatment selection for RA.

## csDMARDs

### Methotrexate: Mechanism, Biomarkers and Alternative Therapy

Despite the wealth of new agents, methotrexate approved by FDA in 1988 remains the primary starting therapy and anchor drug for the treatment of RA, owing to its inexpensive cost, extended safety record, and weekly treatment regimen (25). Mechanism of action in methotrexate is not fully understood. DMARD activity of methotrexate is thought to be due to its polyglutamated form and several mechanisms have been proposed to explain the clinical efficacy in RA, including generation of reactive oxygen species, antagonism of folate-dependent processes, inhibition of methyl-donor production, downregulation of adhesion-molecule, eicosanoids and matrix metalloproteinases (MMPs) expression, modification of cytokine profiles, stimulation of adenosine signaling, inhibition of RANKL/RANK/OPG and JAK/STAT pathways (26–28). Clinical trials with methotrexate monotherapy demonstrate that only 40% of patients with early RA obtain a good response based on ACR50 criteria (29).

Currently, adenosine signaling carries the most robust data for the action of methotrexate in RA (30). Both adenosine A2A receptor (ADORA2A) and A3 receptor (ADORA3) are required for the anti-inflammatory effects of methotrexate (31). The expression of ADORA2A and ADORA3 is increased on immune cells and inversely correlated with disease activity in RA patients (32). It is possible that RA patients with low expression of adenosine receptors will be less responsive to methotrexate. In a study with methotrexate monotherapy, RA patients were categorized into three groups, i.e. good, moderate and nonresponders. A low level of baseline *ADORA3* mRNA expression in blood is associated with nonresponse to methotrexate and could serve as a potential biomarker for distinguishing response to methotrexate therapy in RA (33). Adenosine signaling through ADORA2A leads to the development of Tregs expressing both CD39 and CD73 that may decrease T cell activation (34, 35). A prospective study found that RA patients who did not respond to methotrexate had lower pretreatment CD39 expression on Tregs than methotrexate-responsive patients or healthy controls, suggesting that low expression of CD39 on Tregs could be a biomarker for identifying methotrexate-resistant RA patients (34). Clinical observations suggested that RA patients who had a high intake of adenosine receptor antagonists (such as caffeine) had impaired methotrexate responsiveness, which was consistent with data from animal models (36, 37). However, conflicting evidence manifested that methotrexate efficacy was not affected by adenosine receptor antagonists (38, 39).

In addition to the adenosine signaling, exploration of pharmacometabolic markers, expression and polymorphisms of genes linked to the action of methotrexate is underway to identify methotrexate-responsive or nonresponsive signatures. In a longitudinal study, an increase in methotrexate polyglutamates in erythrocytes was associated with lower disease activity of RA and thought to be a tool for monitoring methotrexate response (40). Changing from oral to subcutaneous methotrexate resulted in increased methotrexate polyglutamates and achieved a better improvement of disease activity of RA (41). A low baseline folate level was associated with a poor response to methotrexate and folate polyglutamate partially antagonized methotrexate efficacy (40, 42). Breast cancer resistance protein [BCRP, gene symbol *ATP Binding Cassette Subfamily G Member 2* (*ABCG2*)] is an ATP-binding cassette efflux transporter that plays an important role in multidrug resistance (43). BCRP transported both methotrexate and polyglutamylated methotrexate and participated in methotrexate resistance (44). Good response to methotrexate was associated with a decrease in expression of BCRP in RA patients (45), while the association of BCRP polymorphisms with the effectiveness of methotrexate was not observed (46), suggesting that BCRP expression was not genetically determined, but might be associated with environmental factors (45). BCRP inhibition could be a strategy for overcoming nonresponse to methotrexate. Consistently, a six-month randomized, double-blind trial enrolling 148 RA patients showed that individuals with partial responses to methotrexate had clinical improvement after combination therapy with an FDA-approved BCRP inhibitor cyclosporine and methotrexate (47). Other studies found that baseline FcγRIIIa expression on CD14+ monocytes was negatively associated to methotrexate response in patients with early RA (48). Circulating miR-10a was upregulated in RA patients with good methotrexate response (49). *Human leukocyte antigen (HLA)-DRB1* shared epitope alleles were linked to a lack of response to methotrexate at the genomic level (50). Stratification based on *HLA-DRB4* allele expression revealed distinct innate and adaptive immune transcriptional patterns in early RA and response to methotrexate therapy could be suggested by a preponderance of innate but not adaptive immune activation (51). A number of single-nucleotide polymorphisms have been investigated for the prediction of methotrexate treatment response. Patients with *solute carrier family 19 A (SLC19A)* rs1051266, *dihydrofolate reductase (DHFR)* rs836788 and *thymidylate synthetase (TYMS)* rs2244500 showed response to methotrexate, while patients with *5-aminoimidazole-4-carboxamide ribonucleotide formyltransferase (ATIC)* rs7563206, *TYMS* rs3786362 and rs2847153 showed reduced effectiveness of methotrexate (52). Patients with *folylpolyglutamate synthetase (FPGS)* rs1544105-AA or -AG and *TYMS* rs2853539-AA genotype were associated with poor response to methotrexate (53). An analysis of the -174 (rs1800795) -GC *IL-6* gene promoter polymorphism in RA patients revealed that genotype -GG may be associated with a poorer response to methotrexate when compared to genotypes -GC and -CC (54), which was in disagreement with another study identifying no association between -GG genotype or G allele and risk of therapeutic failure using different measures for defining response to therapy (55) (**Table 1**).

**Table 1:** Potential biomarkers for response or partial response/nonresponse to csDMARDs.

csDMARDs	Biomarkers for response	Sample size	Reference	Biomarkers for partial response/nonresponse	Sample size	Reference
Methotrexate	Increased methotrexate polyglutamates in erythrocytes	285	(40)	Low level of baseline ADORA3 mRNA expression in blood	100	(33)
	A decrease in expression of BCRP	24	(45)	Lower pretreatment expression of CD39 on Tregs	122	(34)
	Upregulated circulating miR-10a	30	(49)	High intake of adenosine receptor antagonists	39	(36)
	Preponderance of innate immune activation	68	(51)	Low baseline folate level	226	(42)
	SLC19A rs1051266, DHFR rs836788, and TYMS rs2244500	35	(52)	Higher baseline FcγRIIIa expression on CD14+ monocytes	38	(48)
				HLA-DRB1 shared epitope alleles	102	(50)
				ATIC rs7563206, TYMS rs3786362 and rs2847153	35	(52)
				FPGS rs1544105-AA or -AG and TYMS rs2853539-AA	281	(53)
				IL-6 rs1800795 -GC	70	(54)
Leflunomide	Higher A77 1726 steady-state plasma concentration	67	(56)	DHODH rs3213422 A allele	147	(57)
	Estrogen receptor 1 rs9340799-rs2234693 A/T haplotype	115	(58)	Estrogen receptor 1 rs9340799-rs2234693 G/C	115	(58)
				IL-6 rs1800795 -GG	96	(55)
				Higher serum baseline CRP level	250	(59)
Sulfasalazine	Higher exosomal miR-328 in plasma	33	(60)	Higher serum P-gp level	151	(61)
	BCRP rs2231142 -AC or -AA genotype	229	(62)	Increased level of BCRP	229	(62)
	Low interferon IFN/IL-4 ratio	11	(63)			
	HLA-B27-positive	132	(64)			
	Low level of soluble IL-2 receptor	195	(63)			

ADORA3, adenosine A3 receptor; ATIC, ribonucleotide formyltransferase; BCRP, breast cancer resistance protein; CRP, C-reactive protein; DHFR, dihydrofolate reductase; DHODH, dihydroorotate dehydrogenase; FPGS, folylpolyglutamate synthetase; HLA, human leukocyte antigen; IFN, interferon; P-gp, P-glycoprotein; SLC19A, solute carrier family 19 A; TYMS, thymidylate synthetase.

In RA patients with treatment failure of methotrexate, combination therapy is an attractive alternative strategy. A series of clinical trials (such as an observational and descriptive CONAART study enrolling 106 RA patients, a 24-week, randomized, double-blind, controlled SLCTR study enrolling 40 patients and a 48-week, randomized, double-blind, placebo-controlled study enrolling 263 patients) showed that leflunomide in combination with methotrexate was effective for RA patients who did not respond to methotrexate (65, 66). In a 12-month, multicenter, randomized, double-blind, placebo-controlled, parallel-design, dose-finding phase II trial, 115, 105, and 119 RA patients with inadequate response to methotrexate were grouped to receive 2 mg/kg abatacept, 10 mg/kg abatacept, and placebo, in addition to continued methotrexate treatment, respectively. Results showed that 10 mg/kg abatacept presented better anti-inflammatory effects than either 2 mg/kg abatacept and placebo (67). In a double-blind, randomized, parallel-arm MUSICA trial enrolling 309 methotrexate nonresponders, patients were randomly assigned to receive either a high dose (20 mg/week) or a low dose (7.5 mg/week) of methotrexate and received open-label adalimumab for 24 weeks. Adalimumab treatment resulted in a rapid improvement in clinical indices in both groups, which is consistent with results from an OPTIMA study (a 78-week, randomized, double-blind, double-period phase 4 trial enrolling 348 methotrexate inadequate responders) (68) and a PREMIER study (a 2-year, randomized, double-blind, placebo-controlled phase 3 trial enrolling 177 methotrexate inadequate responders) (68). In a prospective, randomized, controlled SURPRISE study enrolling 223 RA patients, tocilizumab in combination with methotrexate more rapidly deceased inflammation than tocilizumab switched from methotrexate, resulting in greater clinical effectiveness and avoidance of joint damage (69).

### Leflunomide: Mechanism, Biomarkers and Alternative Therapy

Leflunomide, approved by the FDA in 1998, is the first choice if methotrexate is contraindicated according to the latest EULAR recommendations (13). Leflunomide acts *via* its active metabolite A77 1726 after the metabolic opening of the isoxazole ring. Its primary target is thought to be dihydroorotate dehydrogenase (DHODH), an enzyme involved in *de novo* pyrimidine production (70). Leflunomide inhibits DHODH activity, resulting in nucleotide depletion, leading to cell cycle arrest and reproduction of rapidly dividing cells, particularly lymphocytes (71). Tyrosine kinases such as Lck and JAK3 in activated T and B cells are also targets of leflunomide (72). Clinical trials have shown that only 40-50% of RA patients taking leflunomide fulfilled the ACR response criteria for a 20% reduction in disease activity (59).

DHODH is located on the inner membrane of mitochondria (73). The human DHODH gene is relatively conserved, with only one common missense polymorphism (rs3213422) in the first exon (19C>A). This polymorphism led to Gln7Lys amino acid substitution in the cationic N-terminal region of the DHODH polypeptide, which was essential for transport and correct insertion into the mitochondrial inner membrane (57). A study reported that RA patients with A allele had a worse response to leflunomide than patients with the C allele (57). A proposed mechanism was that the amino acid substitution generated by the missense polymorphism in *DHODH* might block its import into mitochondria and subsequently affect the action of leflunomide (57). However, another study did not replicate the association between leflunomide response and rs3213422 in a smaller cohort of indviduals (74). Cytochromes P450 (CYP) enzymes, including CYP1A2 and CYP2C19, may be implicated in the conversion of leflunomide to A77 1726. Better response to leflunomide was accompanied by higher A77 1726 steady-state plasma concentration, which was influenced by CYP2C19*2 allele rather than *CYP1A2* polymorphism (56). Evidence demonstrated that the efficacy of DMARDs is more effective in men than in women and estrogens play important roles in the immune response. A study found that the A/T haplotype of the *estrogen receptor 1 (ESR1)* rs9340799-rs2234693 was related with a better sensitivity to leflunomide, while the G/C haplotype was associated with a worse response (58). Researchers also evaluated the influence of the rs1800795-GC *IL-6* gene promoter polymorphism on the therapeutic failure of leflunomide (54, 55, 75). RA patients with *IL-6* rs1800795-GG genotype had a higher risk of failure in therapeutic response to leflunomide when compared to patients with -GC (55), which was contrary to other observations of noninfluence of the rs1800795-GC *IL-6* gene polymorphism on response to leflunomide that used different measures for defining response to therapy (54, 75). Similar to that of methotrexate, drug efflux transporter BCRP was reported to interact with leflunomide and A771726, and an increased level of BCRP might contribute to inadequate response to leflunomide (76). In a 12-month open, prospective trial enrolling 106 RA patients, the combination of a BCRP inhibitor cyclosporine and leflunomide provided statistically significant benefit (77), suggesting that BCRP inhibition could be a potential approach for improving the nonresponse to leflunomide.

Recently, our group found that RA individuals with limited efficacy of leflunomide could be distinguished by higher serum baseline CRP level. Besides the immunomodulation *via* A77 1726, we revealed that leflunomide itself induced aryl hydrocarbon receptor (AHR)-AHR nuclear translocator (ARNT) interaction to inhibit hepatic CRP production and attenuate bone erosion in arthritic rat models. Nevertheless, enforced CRP expression upregulated hypoxia-inducible factor 1α (HIF1α), which competed with AHR for ARNT association and interfered leflunomide-AHR-CRP signaling, leading to nonresponse to leflunomide in arthritic rat models. Hepatocyte-specific HIF1α deletion or an FDA-approved HIF1α inhibitor Acriflavine re-activated leflunomide-AHR-CRP signaling to inhibit bone erosion in leflunomide-nonresponsive animals. This study presented a precision medicine-based therapeutic strategy for overcoming nonresponse to leflunomide in RA (59). In addition, we also performed a 48-week, randomized, controlled clinical trial enrolling 123 RA patients, and showed that leflunomide combined with ligustrazine extracted the Chinese herb Chuanxiong, which was an approved drug in China and had the capacity to inhibit HIF1α expression (78), could significantly reduce disease activity (79). Regarding other alternative treatment options, leflunomide plus infliximab present a general improvement in disease control compared with leflunomide alone in an open, multicenter, retrospective study (80). In a 24 week, double-blind phase of the multicenter, international RELIEF study enrolling 106 inadequate responders to leflunomide, the trend of benefit was indicated for combining leflunomide with sulfasalazine compared with switching to sulfasalazine alone (81) (**Table 1**).

### Sulfasalazine: Mechanism, Biomarkers and Alternative Therapy

Based on the latest EULAR recommendation, sulfasalazine approved by FDA in 1996 is also considered as part of the (first) treatment strategy in RA patients with a contraindication to methotrexate, which is in parallel with leflunomide (13). Among the above agents, sulfasalazine has an acceptable safety profile during pregnancy (82, 83). The mechanism of sulfasalazine is not entirely understood. It is unknown if sulfasalazine or its metabolites such as sulfapyridine and 5-aminosalicylic acid have a role in its anti-inflammatory actions. It is suggested that sulfasalazine inhibits TNF expression by suppressing NF-κB and by inducing caspase 8-induced apoptosis in macrophages (84). Sulfasalazine inhibits osteoclast formation by suppression of RANKL and stimulation of osteoprotegerin (85). Sulfasalazine induces the conversion of adenine nucleotides to adenosine (86). Sulfapyridine and 5-aminosalicylic acid inhibit B cell function and suppress the production of IgM and IgG (87). Sulfapyridine inhibits chemokines IL-8, growth-related gene product-alpha (gro alpha), and monocyte chemotactic protein-1 (MCP-1/CCL2) (88). 5-aminosalicylic acid (5-ASA) inhibits NF-κB signaling by inducing phosphorylation and activation of adenosine monophosphate-activated protein kinase (89). Studies have confirmed that the ACR20 response in RA patients does not exceed 50% after 6 months of sulfasalazine treatment (90, 91).

Research showed that sulfasalazine also interacted primarily with the above-mentioned drug efflux transporter BCRP. BCRP knockout mice had a more than 100-folds increase in plasma concentration of sulfasalazine compared with wild-type (WT) mice (62). Sulfasalazine bioavailability in BCRP knockout mice was 97% compared to 3% in WT mice (92). Of note, treatment of WT mice with a BCRP inhibitor (gefitinib) resulted in a significant increase in plasma concentration and bioavailability of sulfasalazine (92). This suggests that BCRP could be a therapeutic target for eliminating nonresponse to sulfasalazine. Another study found a circulating intestine-derived exosomal miR-328 in plasma, which negatively regulated BCRP expression and resulted in a high plasma concentration of sulfasalazine (60), could be a biomarker of sulfasalazine responsiveness. P-glycoprotein (P-gp), like BCRP, is another drug efflux transporter. It was reported that serum P-gp level was higher in patients with active RA compared to inactive RA patients (93). Serum P-gp level was negatively correlated with sulfasalazine efficacy (61). Sulfasalazine oral bioavailability was markedly increased 2-3 folds in P-gp knockout rats (94). P-gp on Th1 cells participated in the drug resistance to sulfasalazine in RA (95). These results inspire a hypothesis that blockage of P-gp may mimic the effectiveness of BCRP inhibition in overcoming the nonresponse to sulfasalazine. However, it was discouraging to observe that a P-gp inhibitor verapamil could not reverse sulfasalazine nonresponse (96). We assumed that BCRP was mainly responsible for efflux transport of sulfasalazine because plasma concentration of sulfasalazine was more significantly increased in BCRP knockout animals (more than 100 folds) when compared to that in P-gp knockout animals (2-3 folds). It is possible that pharmacological inhibition of P-gp by verapamil could be compensated by the powerful BCRP function in the efflux of sulfasalazine, which should be verified in future studies. Regarding the gene polymorphism, an association between *ABCG2* genotype and remission was found, and carriers of the loss of function alleles (that is, *ABCG2* rs2231142 -AC or -AA genotype) had higher plasma sulfasalazine concentrations (62). Other reports indicated that a low interferon (IFN)/IL-4 ratio is associated with a better response to sulfasalazine (63). HLA-B27-positive patients presented a better response to sulfasalazine (64). A low level of soluble IL-2 receptor predicted remission in early RA patients treated with sulfasalazine (63). Sulfasalazine responders had lower serum MMP-3 values compared to partial responders or nonresponders (97) (**Table 1**).

In an 18-month, randomized, double-blind, placebo-controlled MASCOT study, 165 RA patients who were nonresponders to 6-month sulfasalazine therapy were grouped to receive methotrexate, sulfasalazine and a combination of sulfasalazine and methotrexate for additional 12 months, respectively. The combination significantly decreased DAS and improved the ACR scores when compared to either drug alone (98). This study, together with other randomized controlled trials, were included in a meta-analysis, which suggested that the addition of methotrexate to sulfasalazine is a therapeutic option in SSZ sulfasalazine failure (99). In a 2-year, double-blind, randomized study, 260 sulfasalazine nonresponders were randomly assigned to etanercept, sulfasalazine and etanercept plus sulfasalazine, respectively. A significant improvement was seen in the group treated with etanercept plus sulfasalazine when compared to the other two groups (100). In a 52-week, multicenter, double-blind, parallel-group trial, a total of 123 DMARDs (including sulfasalazine) nonresponders were randomized to receive tacrolimus (an inhibitor targeting calcineurin, which is involved in the production of IL-2) or placebo. Data showed that tacrolimus was helpful for achieving a better clinical response according to ACR20 and EULAR response criteria (101).

## bDMARDs Targeting TNF

### Infliximab: Mechanism, Biomarkers and Alternative Therapy

TNF has been identified its central role in RA at the end of the last century. At the time, little was known about the mechanisms of csDMARDs and people had no better choice for treating RA. This led to a question about whether blockade of TNF could serve as a treatment method. With the development of monoclonal antibodies, this question was firstly answered. In 1992, cA2, now known as infliximab, was produced to confirm that the inflammation driving RA could be suppressed by TNF blockade (102). Infliximab was approved by the FDA in 1999 for RA treatment and attracted an inordinate amount of attention over the past several decades (103). According to the most recent EULAR recommendation, if the treatment goal is not met with the initial csDMARD strategy and there are poor prognostic factors, a bDMARD should be added (13). TNF inhibitors are now the most frequently used bDMARDs (104) and infliximab serves as a first-in-class TNF inhibitor (103). Infliximab is an intravenous administrated, chimeric monoclonal IgG1κ antibody composed of human constant (75%) and murine variable (25%) regions (105). Infliximab binds to both soluble and transmembrane forms of TNF with high affinity, inducing the downregulation of local and systemic pro-inflammatory cytokines (e.g., IL-6), the reduction of lymphocyte and leukocyte migration to sites of inflammation, the induction of apoptosis in TNF-producing cells and the reduction of levels of endothelial adhesion molecules and APR (105). Only approximately 50% of RA patients showed ACR20 response after receiving infliximab treatment (106, 107).

Anti-drug antibodies (ADAs), generated by a T-cell dependent or independent B cell activation pathway, primarily contribute to a poor clinical outcome of biological treatment (108–110). In fact, there are two types of ADAs that can be produced: non-neutralizing antibodies that bind to the medication alongside TNF and neutralizing antibodies that compete with TNF for the antigen-binding site (paratope). Neutralizing antibodies can therefore immediately inhibit the working mechanism of the anti-TNF agents (111). Several clinical trials demonstrated that ADAs might be associated with treatment failure of infliximab (112, 113). Over 40% of patients treated with infliximab developed ADAs (114). Of interest, it was reported that concomitant administration of csDMARDs such as methotrexate might decrease ADAs and prolong therapeutic efficacy (115). Furthermore, the emergence of ADAs may be related with lower serum concentrations of (free) infliximab and a lower clinical response (116). Low infliximab serum concentrations, even 2 months after treatment commencement, were associated with the production of ADAs and predicted later treatment failure (117). However, another study found that lack of response could be due to a lack of infliximab, rather than the presence of ADAs (118), which in line with a hypothesis that drug tolerance was not directly related to the quantity of anti-drug antibodies, but rather depended on the size of the response in relation to the amount of drug that could be neutralized (115).

S100 calcium-binding protein A4 (S100A4) is a metastasis-inducing protein, which promotes the inflammatory response of mononuclear cells *via* the Toll-like receptors (TLR4) signaling in RA (119). It was reported that high S100A4 level was associated with inadequate response to infliximab, ADAs production and high levels of survivin and FMS-like tyrosine kinase 3 (Flt3) ligand (120). Flt3 ligand is a differentiation factor that has predictive value in the preclinical diagnosis of RA (121). Survivin is a downstream molecule of Flt3 signaling and high survivin level predicted poor clinical response to infliximab in RA patients (121, 122). It was proposed that S100A4, survivin and Flt3 ligand could form a new cluster of predictive biomarkers for infliximab nonresponders (120). Another study developed a customized low-density microarray for monitoring mRNA expression in peripheral blood cells, which was helpful for identifying a unique set of genes with differential expressions in infliximab responders and nonresponders. It was important to note that TNF-α itself did not differ significantly between responders and nonresponders, while a clear difference was observed in the kinetics of IFN-related genes during infliximab treatment between the two groups. Specifically, there was sustained inhibition of the IFN signature in responders and reappearance of the signature in nonresponders during infliximab treatment. The underlying mechanism remains to be clarified, and such knowledge will likely identify new therapeutic targets for RA (123). In addition, other data showed that AP-1-associated adaptor complex subunit responsible for protein transport between membrane compartments in receptor-mediated endocytosis was significantly downregulated in peripheral mononuclear cells of infliximab nonresponders (123). TNF receptor recycling was inhibited in nonresponders to infliximab (123). Human immunoglobulin allotypes in the IgG_1_ heavy chain (G_1_m1 and G_1_m17 allotypes) were associated with response to infliximab (124). A significantly decreased CRP level was a predictor of good response with infliximab treatment (125). Patients with low-affinity homozygotes, *fragment crystallizable (Fc) fragment of IgG receptor (FCGR)2A* and *FCGR3A* alleles showed better response to infliximab (126). The baseline level of IgG antibodies against centromere protein F was significantly increased in infliximab-responders (127). In patients with early RA, infliximab < 0.2 μg/mL, and ADAs development were associated with treatment failure and were more common in females (112). TNF level in the intimal lining layer and synovial sublining and number of macrophages, macrophage subsets and T cells were significantly higher in responders than in nonresponders (128). Infliximab responders had a higher number of CD4+CD25+ T cells than nonresponders at baseline (129).

For the gene polymorphism, a series of studies showed that RA patients carrying *TNF-α* rs1800629 -GG genotype were better responders to infliximab, while the presence of A allele significantly decreases the response to infliximab (130–133). *TNF receptor superfamily member 1B* (*TNFRSF1B*, codes TNF receptors 2 (TNFR2)) rs1061622-GG or -TG was related to a lower responsiveness to infliximab (134, 135), while -TT genotype of the *TNFRSF1B* rs1061622 was a predictor of good response to infliximab (136, 137). A possible explanation was that the rs1061622 T>G induced an amino acid substitution at codon 196 (M196R), which located in the fourth cysteine-rich domain of the extracellular region of TNFR2. The R allele elicited a high inflammatory response *via* the TNF- pathway, which could explain the poor response to anti-TNF medication (134). Studies also confirmed that patients with *TNFRSF1B* rs3397-CC and *TNFRSF1B* rs1061631-AA genotypes had an increased risk for nonresponse to infliximab (135). *TNF receptor superfamily member 1A (TNFRSF1A*, codes TNF receptors 1 (TNFR1)) rs767455-AA genotype was associated with a worse EULAR response than -AG or -GG genotype (138). RA patients with homozygous rs396991 polymorphism (V158F) in *FCGR3A* had good response to infliximab (139) (**Table 2**).

**Table 2:** Potential biomarkers for response or partial response/nonresponse to bDMARDs targeting TNF.

bDMARDs	Biomarkers for response	Sample size	Reference	Biomarkers for partial response/nonresponse	Sample size	Reference
Infliximab	Sustained inhibition of the IFN signature	18	(123)	ADAs production	128;26;69	(112, 113, 117)
	G_1_m1 and G_1_m17 allotypes	1037	(124)	Lack of infliximab	94	(118)
	Decreased CRP level	207	(125)	High S100A4 level	87	(120)
	Patients with low-affinity homozygotes, Fc fragment of FCGR2A and FCGR3A alleles	91	(126)	High survivin level	87	(122)
	Increased baseline level of IgG antibodies against centromere protein F	185	(127)	Downregulated AP-1-associated adaptor complex subunit	18	(123)
	Higher TNF level and number of macrophages, macrophage subsets and T cells	143	(128)	TNF receptor recycling was inhibited	18	(123)
	A higher number of CD4+CD25+ T cells at baseline	44	(129)	Infliximab < 0.2 μg/mL, and ADAs development	128	(112)
	TNF-α rs1800629 -GG	59;54	(130, 131)	TNF-α rs1800629 A allele	54;692	(131, 133)
	TNF-α rs1800629 G alleles	2127	(132)	TNFRSF1B rs1061622-GG or -TG	148;2637	(134, 135)
	TNFRSF1B rs1061622-TT	175;105	(136, 137)	TNFRSF1B rs3397-CC and TNFRSF1B rs1061631-AA	2637	(135)
	FCGR3A homozygous rs396991	41	(139)	TNFRSF1A rs767455-AA	280	(138)
Etanercept	TNF-α rs1800629-GG	86;86	(140, 141)	NUBPL rs2378945 A allele	755	(142)
	TNF-α rs1799724-TT or -CT	280	(138)	IL-10 promoter microsatellite allele IL10.G13	50	(143)
	TNFRSF1A rs767455-AA	280	(138)	Combination of TGF-β1 codon 25 C and IL-1RN intron 2 A2 allele	123	(144)
	TNFRSF1B rs1061622-TT	175	(136)	More methylated 4 CpG within exon 7 of LRPAP1	72	(145)
	TNFRSF1B rs1061622-TT	105	(137)			
	The combination of TNF-α rs1800629-GG and IL-10 rs1800896-GG	123	(144)			
	IL-6 rs1800795-GG	73;77;199	(146–148)			
	low TNF-α and IL-6 production	73	(146)			
	IL-10 promoter microsatellite allele IL10.R3 and the haplotype R3-G9	50	(143)			
	Higher expression of CD84	2706	(149)			
	Increased isoleucine, leucine, valine, alanine, glutamine	27	(150)			
	Increased tyrosine, glucose and 3-hydroxybutyrate	27	(150)			
	Higher baseline serum CRP, IL-1β and IL-17A	128	(151)			
Adalimumab	Finer ACPA specificities in ACPA-negative	286	(152)	Carrying the same IgG allotype as present on the adalimumab IgG	250	(153, 154)
	ACPA positive	646	(155)	< 9.4% of SIRPα/β-expressing memory B cells	57	(156)
	Decreased CD68 and MMP-3 expression in the synovium	5	(157)	The presence of ACPA	642	(158)
	Lower chemokine receptor 6 expression	48	(159)	Elevated baseline NLR and PLR	410	(160)
	Increased Th17 and Th1	48	(159)	TNF-α rs1800629 G haplotype in a homozygous form	388	(161)
	Elevated baseline CXCL10 and CXCL13	29	(162)			
	Increased expression of CD11c	27	(163)			
	Higher MRP 8/14	170	(164)			
	High sICAM1 and low CXCL13	69	(165)			
	TNF-α rs1800629-GG	81	(166)			
	TNF-α rs1799724-TT	280	(138)			
	TNFR1 rs767455-AA	280	(138)			
	TNFR2 676 rs1061622-TT	105	(137)			
	Low-affinity FCGR2A-R(A)* allele	302	(167)			
	IL-6 rs1800795-GG	199	(168)			
Certolizumab pegol	No Data	N/A	N/A	Low drug concentration	40	(169)
				Early response to certolizumab pegol	198	(170)
				Failure to achieve improvement in DAS28 within the first 3 months of therapy	783	(171)
				Failure to achieve improvements in DAS28(ESR) within the first 3 months	955	(172)
				Failure to achieve improvements in SJC or CDAI within the first 3 months	955	(172)
				CDAI nonresponse at 3 months	574	(173)
				High serum pretreatment ratio of type I IFNβ/α (> 1.3) or undetectable type I IFN	124;15	(174, 175)
Golimumab	Golimumab concentration ≥ 1.0 mg/L	91	(176)	Sustained increase of IL-6, CRP, IL-2 receptor alpha chain, and MMP-1	138	(177)
	Decreased in serum amyloid A, E-selectin and MMP-9	137	(178)			
	Lower HSQ, ESR (or CRP) and TJC (or SJC) scores	3280	(179)			
	Being male, younger age, and absence of comorbidities	3280	(179)			

ACPA, Anti-citrullinated protein antibodie; ADAs, anti-drug antibodies; CD, Cluster of Differentiation; CDAI, Clinical Disease Activity Index; CRP, C-reactive protein; CXCL, C-X-C motif chemokine ligand 10; DAS, disease activity score; ESR, erythrocyte sedimentation rate; FCGR, Fc fragment of IgG receptor; HSQ, Health Status Questionnaire; IFN, interferon; IL-1RN, Interleukin 1 receptor antagonist; IL-2R, interleukin-2 receptor subunit; LRPAP1, LDL Receptor Related Protein Associated Protein 1; MMP, matrix metallopeptidase; MRP8/14, Myeloid-related protein 8/14; NLR, neutrophil-to-lymphocyte ratio; NUBPL, Nucleotide Binding Protein Like; PLR, platelet-to-lymphocyte ratio; RF, rheumatoid factor; SIRPα/β, Signal regulatory protein α/β; SJC, swollen joint count; Th, T helper; TJC, tender joint count; TNF, Tumor necrosis factor; TNFRSF, tumor necrosis factor receptor superfamily.

In a cohort study, in the context of methotrexate, 95 consecutive patients with RA who were first treated with infliximab were switched to etanercept due to a lack of response (either primary, secondary, or with toxicity). Significant DAS28 reductions and ACR response were reported in the overall cohort and nonresponse subtype groups after 12 weeks of medication. Sixty-one percent of the group received a moderate or good EULAR score, confirming that etanercept was successful in patients who did not respond to infliximab (180). In a 12-week, multicenter, open-label clinical study involving 6610 difficult-to-treat patients using DMARDs (including 11% infliximab), results showed that adalimumab alone or in combination with standard DMARDs was effective to improving ACR20 response and EULAR response (181). In a 12-week, double-blind period of the phase IIIb trial, 1063 DMARDs nonresponders (37.6% had previous TNF inhibitor use including infliximab) were randomized to certolizumab pegol or placebo. Certolizumab pegol was linked to faster and more consistent clinical responses as well as increased physical function (182). In a prospective, 12-week, open label, single-arm, observational trial, 25 patients were enrolled, 18 of whom had stopped taking infliximab due to inefficacy, and 22 who had completed 12 weeks of switching etanercept medication. After 12 weeks, 64% of patients had an ACR20 response (183).

### Etanercept: Mechanism, Biomarkers and Alternative Therapy

Etanercept was originally developed for treating sepsis but failed in clinical trials. It was then tested for treating RA (184). Etanercept was approved by FDA in 1998 (185), one year ahead of infliximab. Etanercept is a soluble fusion protein consisting of two human 75 kD TNFR 2, each linked to an Fc portion of human IgG_1_ (186). Functioning as a decoy receptor, etanercept binds to both TNF-α and TNF-β with much greater affinity than endogenous soluble TNFRs, which is unique from other TNF inhibitors that are variants of anti-TNF antibodies (187). TNF inhibition with etanercept modifies various physiologic responses caused or regulated by TNF, including the expression of adhesion molecules involved in leukocyte migration, serum levels of cytokines (e.g., IL-6), and serum levels of MMP-3 (188–190). Only approximately 40% patients achieved an ACR50 response when treated by etanercept monotherapy (191, 192).

ADAs against etanercept were not consistently detected (193, 194) and had no relationship with reduced clinical response (195, 196), while low etanercept level was associated with nonresponse (197).This could be explained by the following two reasons. Firstly, etanercept formed smaller immune complexes compared to infliximab when bound to TNF, which might reduce uptake by antigen-presenting cells (198). Secondly, only the fusion part of the etanercept protein contained foreign epitopes while the TNF binding area did not, which led to low immunogenicity (197). Same as infliximab, *TNF-α* rs1800629-GG was associated with better response to etanercept than -AA or -AG (140, 141). *TNF-α* rs1799724-TT or -CT were associated with better response than -CC (199). *TNF-α* rs1799724-TT showed better response than C allele carriers (138). *TNFRSF1A* rs767455-AA was associated with better response than -GG (138); *TNFRSF1B* rs1061622-TT was associated with better response to etanercept (136, 137). A combination of alleles (*TNF-α* rs1800629-GG and *IL-10* rs1800896-GG) was associated with good response to etanercept (144). Other polymorphisms were also contributory. Several studies have confirmed that *IL-6* rs1800795-GG was associated with better response than -GC or -CC (146–148). Patients with the combination of *IL-6* rs1800795-GG and *TNF-α* rs1800629-GG genotype were more frequent among the responders compared to those with other combined genotypes. Patients with low TNF- and IL-6 production were the best responders to etanercept therapy (146). *Nucleotide-binding protein-like (NUBPL)* rs2378945 minor allele (A) had a significant association with poor response to etanercept (142). The *IL-10* promoter microsatellite allele IL10.R3 and the haplotype R3-G9 were considerably more prevalent in patients who responded well to etanercept, whereas IL10.G13 was more common in patients who responded moderately or no response (143).

A combination of C allele in codon 25 of the *transforming growth factor beta 1 (TGF-β1)* gene and the A2 allele in intron 2 of the *interleukin 1 receptor antagonist* (*IL-1RN*, codes IL-1Ra) gene, were associated with nonresponse to etanercept (144). On one hand, TGF-1 has been shown to suppress T-cell functions such as proliferation and differentiation of cytotoxic T-cells and T-helper cells (200). The homozygous genotype CC, whether at codon 10 or codon 25, was highly related with reduced TGF-1 production (201). On the other hand, patients carrying the *IL-1RN* A2 allele had increased production of IL-1 and possibly decreased IL-1Ra (202–204). It was the above two reasons that contributed to nonresponse to etanercept. In addition, 4 CpG within exon 7 of *LDL receptor related protein associated protein 1 (LRPAP1)* were observed to be more methylated in nonresponders (145). LRPAP1 is a receptor for TGF-β1 (205). We speculated that *LRPAP1* methylation blocked the function of TGF-β1 and then induced etanercept nonresponse. Thus, DNA methylation inhibitor might be helpful for such patients (206). Moreover, higher expression of CD84 was associated with better response to etanercept (149). Increased isoleucine, leucine, valine, alanine, glutamine, tyrosine, glucose and 3-hydroxybutyrate levels were associated with good response to etanercept (150). Higher baseline serum CRP, IL-1β and IL-17A were associated with better response to etanercept (151) (**Table 2**).

In an open-label, single-blind clinical trial, 28 patients with an inadequate response to etanercept were randomized 1:1 to receive infliximab, or to continue etanercept, with background methotrexate treatment. At week 16, 62% of infliximab-treated patients had ACR20 responses, compared to 29% of etanercept-treated patients (207). A multicenter, randomized, double-blind, placebo-controlled, phase III trial enrolled 461 patients with nonresponse to TNF inhibitors (including etanercept). Patients with continued background csDMARDs treatment were assigned in a 1:1:1 ratio to receive subcutaneous injections of placebo, 50 mg golimumab, or 100 mg golimumab. 140 patients achieved ACR20 at week 14, including 18% patients on placebo, 35% patients on 50 mg golimumab, and 38% patients on 100 mg golimumab, suggesting golimumab was a good choice for patients who had previously received one or more TNF-α inhibitors (208). These results were confirmed in a long-term extension, multicenter, randomized, double-blind, placebo-controlled, phase 3 GO-AFTER study with up to five years of therapy (209). Another trial included 18 RA patients who were first treated with etanercept and subsequently switched to infliximab due to inefficacy. The mean best DAS28 after switching to infliximab was considerably better than the previous result, indicating that a trial of infliximab was reasonable for such patients (210).

### Adalimumab: Mechanism, Biomarkers and Alternative Therapy

Adalimumab, approved by FDA in 2002, is the first fully human, high-affinity, recombinant IgG_1_ anti-TNF monoclonal antibody (211). It has high selectivity and affinity for TNF, a low degree of immunogenicity, and a half-life comparable to that of IgG_1_, allowing every-other-week dosing for patients (211). Adalimumab exerts its therapeutic effects by blocking the interaction of TNF with the p55 and p75 cell surface TNFR (211). By blocking TNF signaling, MMP-1 and MMP-3 are downregulated and osteoclast maturation and activation are inhibited (190, 212). Only approximately 40% of RA patients showed ACR50 response after receiving adalimumab monotherapy (213).

ADAs rate of adalimumab was 28% and RA patients carrying the same IgG allotype as present on the adalimumab IgG had a high frequency of ADAs (153, 154), suggesting that these patients might not gain substantial clinical benefit from adalimumab treatment. In addition, a frequency of < 9.4% of signal regulatory protein (SIRP)α/β-expressing memory B cells predicted RA patients that would develop ADAs, and consequentially failed to respond to treatment (156). It was postulated that evaluating the percentage of SIRP/-expressing memory B cells in patients prior to adalimumab treatment could be a valuable biomarker for identifying a subset of active RA patients who will develop ADAs and develop nonresponse to adalimumab (156). Interestingly, there was no functional data showing the role of SIRP in B cells, while SIRPα was reported to be a critical regulator of myeloid cell activation *via* binding to CD47 and SIRPα/CD47 axis limited the efficacy of tumor-opsonising antibodies (214). Thus, it is necessary to explore the underlying mechanism involving SIRPα/β-expressing memory B cells in ADAs response of adalimumab. In addition to SIRPα/β-expressing memory B cells, it was suggested that the existence of other specific risk factors, genetic or environmental, predisposed some individuals to develop adalimumab ADAs (156). For example, smoking could predict ADAs development (215) and RA patients with over 1 year disease duration or with an initial DAS28 over 3.2 have a higher risk of ADAs positivity (215). In addition to ADAs, the status of anti-citrullinated protein/peptide antibody (ACPA) could affect the efficacy of adalimumab. Finer ACPA specificities in ACPA-negative might be predictive of response to treatment (adalimumab or methotrexate) (152) and adalimumab was more effective in patients who were ACPA positive than in those who were ACPA negative at baseline (155). However, another study reported a contradictory result that the presence of ACPA was associated with a reduced response to TNF inhibitors including adalimumab (158). Decreased CD68 and MMP-3 expression in the synovium was associated with a good response to adalimumab (157). RA patients with response to adalimumab had significantly lower chemokine receptor 6 (CCR6) expression and increased Th17 and Th1 (159). Elevated baseline levels of chemokine (C-X-C motif) ligand (CXCL)10 and CXCL13 were associated with favorable response to adalimumab (162). Increased expression of CD11c was correlated with a good response to adalimumab (163). Higher myeloid-related protein (MRP)8/14 levels predicted good adalimumab response (164). Elevated baseline neutrophil-to-lymphocyte ratio (NLR) and platelet-to-lymphocyte ratio (PLR) were associated with a higher risk of nonresponse to adalimumab (160). RA patients with high soluble intercellular adhesion molecule 1 (sICAM1) and low CXCL13 had a good clinical response to adalimumab (165) (**Table 2**).

Polymorphisms of TNF and TNFR also influenced response to adalimumab. A study showed that *TNF-α* rs1800629-GG was associated with a better response to adalimumab than -GA or -AA (166). In contrast, another study reported that *TNF-α* rs1800629 G haplotype in a homozygous form was associated with a lower response (161). Two meta-analyses failed to demonstrate that the rs1800629 G/A genotype, whether heterozygous or homozygous, is linked to a poor response to anti-TNF medication treatment (216, 217). In *TNF-α* rs1800629-GG patients, ACPA status did not affect the clinical response to adalimumab (218). *TNF-α* rs1799724-TT showed a better response than C allele carriers with adalimumab treatment (138). *TNFR1* 36 (rs767455) -AA was associated with a better response to adalimumab than -GG (138); RA patients with *TNFR2* 676 (rs1061622) -TT demonstrated a better response compared to those with -TG (137). Other polymorphisms were also studied. When RA patients treated with adalimumab, low-affinity *Fc gamma receptors 2A (FCGR2A)*-R(A)* allele shows a better EULAR good response (167), *IL-6* rs1800795-GG was associated with a better response than -GC or -CC (168).

In a 52-week, double-blind, placebo-controlled, active-controlled phase III study, 1305 patients were randomized 3:3:2 to placebo, baricitinib (a JAK inhibitor) or adalimumab. At week 16, adalimumab nonresponders received rescue treatment with baricitinib. Results showed that switching from adalimumab to baricitinib was associated with improvements in disease management, physical function, and pain (219). In a 24-weeks Single-Arm study, 90 patients discontinued prior adalimumab treatment and continued methotrexate combined with etanercept for 24 weeks. ACR response data demonstrated that switching to etanercept was a therapeutic option in patients with RA who failed adalimumab treatment. ADAs response was examined to explain the treatment failure in this study. It was shown that patients with nonresponse to adalimumab produced higher anti-adalimumab antibodies, which did not cross-react with etanercept and provided additional support for switching to etanercept (220). In a 48-week, randomized, double-blind, SELECT-COMPARE study, 1629 patients were grouped 2:2:1 to upadacitinib (a JAK inhibitor), placebo or adalimumab, with stable background methotrexate. Upadacitinib in combination with methotrexate demonstrated superior clinical and functional responses versus adalimumab combined with methotrexate (221). Patients who did not respond well to adalimumab saw clinically significant improvements after switching to upadacitinib (221).

### Certolizumab Pegol: Mechanism, Biomarkers and Alternative Therapy

Certolizumab pegol, approved by FDA in 2009, is an antigen-binding fragment (Fab) of a recombinant humanized monoclonal antibody conjugated to PEG. PEGylation enables the increase of the plasma half-life and solubility and reduces the immunogenicity and protease sensitivity (222). Certolizumab pegol binds to TNF with greater affinity and is more effective than adalimumab and infliximab at neutralizing soluble TNF-mediated signaling, but has equal or lesser potency than etanercept (223). Certolizumab pegol may be more effective in penetrating inflamed arthritic tissue than other anti-TNF medications and it cannot be actively transported through the placenta during pregnancy (222). ACR20 and ACR50 response in RA patients was only about 45% and 23% after 6-month treatment of certolizumab pegol, respectively (224).

Like other biologic agents, certolizumab pegol elicited immunogenic response, resulting in the formation of ADAs with a high incidence of about 65% (169). Further research showed that >97% of ADAs to certolizumab pegol was directed to the paratope of the drug and were thus neutralizing, indicating these patients with neutralizing ADAs had especially higher risk for drug nonresponse (225). However, a recent study advocated not to overvalue ADAs in a clinical setting, unless certolizumab pegol concentration was low, as they found that the drug concentration but not the presence of ADAs was highly correlated with the capacity to neutralize TNF (169). Clinimetric measurements were found to be associated with nonresponse to certolizumab pegol during course of treatment. Early response to certolizumab pegol predicted long-term outcomes (170). Failure to achieve improvement in DAS28 within the first 3 months of therapy was predictive of a low probability of achieving low disease activity at 12 months using certolizumab pegol (171). Failure to achieve improvements in DAS28(ESR), SJC or CDAI within the first 3 months of certolizumab pegol therapy was associated with a low chance of achieving low disease activity at 7 months (172). CDAI nonresponse at 3 months was a predictor of failure to achieve the therapeutic target of low disease activity at 12 months in patients with RA initiating treatment with certolizumab pegol (173). Studies observed that RA patients who had high serum pretreatment ratio of type I IFNβ/α (> 1.3) or undetectable type I IFN were likely to have EULAR nonresponse to TNF inhibitors (including certolizumab pegol) (174, 175). Mechanically, the pattern of gene expression that differed between the response and nonresponse groups suggested that canonical type I IFN pathway signaling *via* JAK/STAT was increased in peripheral blood classical monocytes of RA patients who were likely to respond to TNF inhibition, whereas JAK/STAT-independent non-canonical IFNβ-IFNAR1 signaling was increased in nonclassical monocytes of those who were not likely to respond to TNF inhibition (175, 226). Notably, JAK1 expression was absent in both classical and nonclassical monocytes from the patients with undetectable IFN or IFNβ/α > 1.3, suggesting JAK1 could be a predictive factor for nonresponders to TNF inhibitors (175) (**Table 2**).

In a 104-week, randomized, single-blind (double-blind until week 12 and investigator blind thereafter), parallel-group, head-to-head superiority study, 457 RA patients were treated by certolizumab pegol plus methotrexate or adalimumab plus methotrexate. At week 12, 65 nonresponders to certolizumab pegol were switched to adalimumab and 57 non-responders to adalimumab were switched to certolizumab pegol. Certolizumab pegol plus methotrexate was not found to be superior to adalimumab plus methotrexate. For patients with primary therapy failure, clinical benefit could be observed after drug switching in both groups (227). In a 2-year, phase 2a, double-blind, proof-of-concept study, 52 RA patients with inadequate response to certolizumab pegol received certolizumab pegol plus bimekizumab (a monoclonal IgG1 antibody that selectively inhibits IL-17A and IL-17F). Data showed that reduction of DAS28 was greater in the group treated with bimekizumab in combination with certolizumab pegol compared with the group treated with certolizumab pegol plus placebo after 20-week treatment (228). This suggested that the add-on therapy of bimekizumab was of great clinical significance for nonresponsive patients to certolizumab pegol.

### Golimumab: Mechanism, Biomarkers and Alternative Therapy

Golimumab, a fully human IgG1κ monoclonal antibody with directed against the soluble and membrane bound forms of TNF-α, was the latest TNF inhibitor approved by the FDA in 2009 (229, 230). As a newer, second-generation TNF inhibitor, the clinical experience of golimumab was less in comparison with the older ones such as infliximab, etanercept and adalimumab (230). Different with other TNF inhibitors, golimumab has a specific mode of action: it binds to a distinct epitope on TNF-α that does not overlap with the binding residues of TNFR2, but the complex sterically hinders TNFR2 as well as TNFR1 from associating with TNF-α (231). In combination with methotrexate, golimumab has a UK marketing authorization for RA therapy (232), which may in part be attributable to the concomitant use of methotrexate reduces the clearance of golimumab by approximately 35% (230). A GO-BEFORE trial enrolling 637 RA patients and a GO-FORWARD trial enrolling 444 RA patients demonstrated that golimumab achieved ACR50 response in approximately 40-50% of patients (233, 234).

Regarding immunogenicity of golimumab, complementarity determining region loop grafting was developed to reduce some of the immunogenic issues associated with chimeric antibodies (235). A study showed that only 6.5% of golimumab-treated patients developed ADAs (236). However, other studies detected 31.7% of ADAs for golimumab using a more sensitive method (237). Some patients showed good response to golimumab even with a presence of ADAs and the numbers of ADAs-positive patients were insufficient to determine whether these ADAs are associated with an impaired clinical response (196). Golimumab concentration ≥ 1.0 mg/L was associated with improved treatment response (176). Larger magnitudes of the decrease in serum amyloid A (SAA), E-selectin and MMP-9 were observed in responders to golimumab plus methotrexate relative to nonresponders (178). Greater likelihood of low disease activity and remission were associated with being male, younger age, lower health assessment questionnaire, ESR (or CRP) and TJC (or SJC) scores and absence of comorbidities in golimumab-treated RA patients (179). Sustained increase of markers including IL-6, CRP, IL-2 receptor alpha chain, and MMP-1, was presented in golimumab-inadequate responders (177) (**Table 2**).

Currently, alternative therapies for golimumab are poorly studied. In a latest study, according to real-life data extracted from 11 Austrian social health insurance funds covering 86% of the Austrian population, 7637 RA patients on bDMARD therapy were retrieved in total. Golimumab was prescribed in 15% RA patients. After golimumab failure, patients were often switched to an IL-6R antagonist tocilizumab and efficacy was waiting to be determined (238).

## bDMARDs Blocking T Cells, CD20 and IL-6R

### Abatacept: Mechanism, Biomarkers and Alternative Therapy

Beyond TNF, CD28 signaling play a key role in T cell process and RA development (239). According to ACR guideline, RA patients with their first TNF Inhibitor failure could switch to abatacept (240). Abatacept, which was approved by the FDA in 2005, is a soluble, recombinant, fully humanized fusion protein that consists of the extracellular domain of cytotoxic T-lymphocyte antigen 4 (CTLA-4) and the Fc portion of IgG1 that has been modified to reduce the Fc region’s capacity to induce antibody-dependent and complement-dependent cytotoxicity (241). Abatacept is the first biological drug to primarily target T-cell activation in RA. Abatacept works therapeutically by binding to the costimulatory molecules CD80 and CD86 on antigen-presenting cells (APCs), preventing them from interacting with CD28 on T cells (241). Abatacept also functions through regulating macrophages (242, 243), monocytes (244) and B cells (245–248). Abatacept significantly decreases expression of IFN-γ, IL-1β, MMP-1 and MMP-3 (247). There are two approved formulations for abatacept, intravenous and subcutaneous, which have similar efficacy and safety profile (249). Immunogenicity for abatacept is low and transient, and do not interfere with clinical response (250). Abatacept can be used in conjunction with csDMARDs or as a monotherapy. However, because of an increased risk of infections and malignancies without a significant improvement in efficacy, concurrent treatment with abatacept and other bDMARDs is not indicated (251). In two phase III trials, patients treated with abatacept achieve ACR20 at 66% (252) and 50% (253), respectively.

Rheumatoid factor (RF) was the first autoantibody to be discovered in RA patients (254). A pooled study of data from 9 observational RA registries in Europe found that RF positivity was related with improved abatacept medication efficacy (255). However, A meta-analysis of clinical trials found that no association was found between abatacept response and RF (256). Recently, RF seropositivity could predict increased abatacept retention and abatacept showed preferential efficacy in patients with high-titer RF (257, 258). ACPA/anti-cyclic citrullinated peptides (anti-CCP, a surrogate for ACPA) (259), added to the 2010 ACR/EULAR diagnostic criteria (260), is a hallmark of RA and plays a role in disease pathogenesis (261). A retrospective observational cohort study found there was significantly higher clinical response and drug retention rate in ACPA-positive patients treated with abatacept (262). In an AMPLE trial, abatacept was more effective in patients who were positive for anti-CCP than those who were negative for anti-CCP at baseline (155). Data from the AVERT trial showed that abatacept patients who were anti-CCP IgM positive at baseline had stronger clinical effectiveness than those who were anti-CCP IgM negative at baseline (263). Patients in clinical trials are often a selected population that may not reflect the diverse population observed in ordinary practice settings. As a result, more real-world data are needed to investigate the relationship between anti-CCP status and abatacept therapy effects. According to real-world data from US clinical practices, better clinical response was observed in anti-CCP positive patients (264), which was consistent with a real-world ACTION study reporting that anti-CCP positive status was associated with greater efficacy of abatacept than seronegative status (257). The real-world ACTION study also discovered that double ACPA/RF positive led in increased abatacept retention rates (265). A major limitation still exists in these studies, i.e., they categorized patients according to ACPA/anti-CCP status (e.g., positive vs. negative) rather than titers. Most recently, RA patients treated with abatacept were classified based on ACPA/anti-CCP titers. Results showed that clinical effect of abatacept was most pronounced in patients with high-titer ACPA (258). However, this seems to be contradicting with other two studies, which found that sustained response to abatacept was associated with an early reduction in ACPA titers (266), and abatacept was more effective in patients who showed decreasing anti-CCP antibody titers during treatment (267). Taken together, although ACPA/anti-CCP has been used as a biomarker of disease progression in RA patients for decades, its exact relationship with abatacept response still needs to be explored.

Furthermore, in RA, a large baseline number of circulating CD28 negative T cells may indicate nonresponse to abatacept (268). RNA elongation, apoptosis-related, and NK cell-specifically expressed genes were upregulated in abatacept nonresponders, while inflammasome genes were upregulated in infliximab nonresponders and B cell-specifically expressed genes were downregulated in tocilizumab nonresponders (269). When RA patients with *CTLA-4* rs5742909 T or *CTLA-4* rs231775 G polymorphisms received abatacept, they had a greater EULAR response and lower disease activity (270). By metabolomic analysis, low level of 3-aminobutyric acid and high levels of quinic acid and citrate were observed in responders to abatacept treatment (271). Serum CXCL10 level was associated with better response to abatacept (272). A higher level of CD24-high and CD27 positive regulatory B cells at baseline was associated with DAS28 remission and a good EULAR response in abatacept-treated patients (273). Reduced type I IFN score, and higher expression of dendritic cells-related genes (*Basic Leucine Zipper ATF-Like Transcription Factor 2 (BATF)*, *Lysosomal Associated Membrane Protein 3 (LAMP-3)*, *CD83*, *C-type Lectin Domain Family 4 Member A (CLEC4A)*, *Indoleamine 2, 3-dioxygenase 1 (IDO)*, *interferon regulatory factor (IRF)7*, *STAT1*, *STAT2* and *TNF Superfamily Member 10 (TNFSF10)*) could be used as biomarkers to predict good response to abatacept (274). Increased dickkopf (Dkk)-1 serum level and sclerostin might indicate a poor prognosis and resistance to abatacept treatment in RA patients (275). Increased cartilage oligomeric matrix protein level served as a strong predictive biomarker for inadequate response to abatacept treatment for RA patients with a first TNF inhibitor failure (276) (**Table 3**).

**Table 3:** Potential biomarkers for response or partial response/nonresponse to bDMARDs blocking T cells, CD20 and IL-6R.

bDMARDs	Biomarkers for response	Sample size	Reference	Biomarkers for partial response/nonresponse	Sample size	Reference
Abatacept	RF positivity	2942	(255)	High circulating CD28 negative T cells at baseline	32	(268)
	RF seropositivity and high-titer RF	2350;40	(257, 258)	Upregulated RNA elongation, apoptosis-related expressed genes	209	(269)
	ACPA-positive	553	(262)	NK cell-specifically expressed genes	209	(269)
	Anti-CCP positive at baseline	646;2281;2350	(155, 257, 264)	Increased dickkopf-1 serum level and sclerostin	59	(275)
	Anti-CCP IgM positive at baseline	511	(263)	Increased cartilage oligomeric matrix protein level	30	(276)
	High-titer ACPA	40	(258)			
	An early reduction in ACPA titers	149	(266)			
	Decreasing anti-CCP antibody titers	109	(267)			
	CTLA-4 rs5742909 T or CTLA-4 rs231775 G	109	(270)			
	Low level 3-aminobutyric acid; high level quinic acid and citrate	43	(271)			
	Decreased serum CXCL10 level	25	(272)			
	Baseline higher level of CD24-high and CD27 positive regulatory B cells	38	(273)			
	Reduced type I IFN score; higher expression of dendritic cells-related genes	168	(274)			
Rituximab	Low or absence of baseline IFN type I response genes expression	226	(277)	Increased CD46 expression in peripheral B cells	10	(278)
	Decreased mTOR, p21, caspase 3, ULK1, TNFα, IL-1β, and cathepsin K	42	(279)	Persistence of switched memory B cells in lymphoid tissues	14	(280)
	Reduction in circulating CD4+ T cell number	33	(281)	ADAs formation	96	(282)
	Depletion of CD19+/-CD27++CD38++ preplasma cells	31	(283)	IL-6 rs1800795-CC	142	(284)
	IL-6 rs1800795-GC or -GG	142	(284)	Incomplete depletion of baseline peripheral blood B cell receptor repertoire	24	(285)
	FCGR2A rs1801274-TT	142	(286)	Fast rebuilding of functional B cells	26	(287)
	FCGR3A rs396991 G allele	142;224;212	(286, 288, 289)	Total lymphocyte >2910/μL combined with plasmablast >2.85% at baseline	44	(290)
	FCGR3A rs396991-GT	177	(291)	Higher circulating preplasma at baseline and incomplete B cell depletion	158	(20)
	Homozygotes BAFF rs9514828 C and rs9514828 T	224	(292)	Persistently high serum IL-6 level	51	(293)
	IRF5 rs2004640-TG	115	(294)			
	TGFβ1 rs1800471-GC or -CT	63	(295)			
	TTTT haplotype in promoter region of B cell stimulator gene	269	(296)			
	Higher initial depth of B cell depletion	180	(297)			
Tocilizumab	IL-6R 12083537-AA	171	(298)	IL-6R rs12083537 A allele and the rs4329505 C allele	79	(299)
	CD69 rs11052877 A alleles	79	(300)	High sICAM1 and low CXCL13 at the synovial level	69	(165)
	FCGR3A rs396991-TT	142	(286)	Higher enrichment of TNF-induced gene transcripts	65	(301)
	RF positivity at baseline	23	(256)			
	High baseline CRP level	204	(302)			
	Soluble IL-6R low at baseline	43	(303)			
	Upregulated gene IFI6, MX2, OASL and one encoding metallothionein-1G	60	(304)			
	Low serum D-dimer and IL-1β levels	65	(305)			
	Pre-treatment serum 14-3-3η levels	149	(306)			
	Increased TRAV8-3, EPHA4, CCDC32, and a decrease of DHFR	13	(307)			
	High soluble gp130Fc	138	(308)			
	Low IL-17A level	88	(309)			
	A higher baseline NK cell count	92	(310)			
	Low sICAM1 and high CXCL13	69	(165)			
Sarilumab	ACPA positive	2108	(311)	No data	N/A	N/A
	RF positive and/or CCP positive	1743	(312)			
	Patients received 200 mg sarilumab every 2 weeks	1743	(312)			
	Elevated baseline level of IL-6	1193	(313)			
	Lower level of sICAM1 at baseline	291	(314)			

ACPA, Anti-citrullinated protein antibodie; ADAs, anti-drug antibodies; CASP3, caspase 3; CCDC32, Coiled-Coil Domain Containing 32; CCP, cyclic citrullinated peptide; CD, Cluster of Differentiation; CDAI, Clinical Disease Activity Index; CRP, C-reactive protein; CTLA-4, cytotoxic T-lymphocyte-associated protein 4; CTSK, cathepsin K; CXCL, The chemokine (C-X-C motif) ligand; DHFR, dihydrofolate reductase; EPHA4, ephrin type-A receptor 4; FCGR, Fc fragment of IgG receptor; HAQ, Health Assessment Questionnaire; IFI6, Interferon Alpha Inducible Protein 6; IFN, interferons; MX2, MX Dynamin Like GTPase 2; NK, Natural killer; OASL, 2’-5’-Oligoadenylate Synthetase Like; RF, Rheumatoid Factor; sICAM1, soluble intercellular adhesion molecule-1; TGFβ1, transforming growth factor beta 1; TNF, Tumor necrosis factor; TRAV, T Cell Receptor Alpha Variable.

In a multicenter, retrospective study, RA patients initially treated with abatacept (n = 76, most of them discontinued abatacept due to lack of effectiveness) were switched to either TNF inhibitors (adalimumab, certolizumab pegol, etanercept, golimumab, infliximab) or tocilizumab. Drug retention was estimated after 24 months. Switching to tocilizumab resulted in higher retention due to efficacy, although total retention was comparable when compared to TNF inhibitors (315). In a retrospective cohort study involving 550 RA patients treated with abatacept, 25 inadequate responders underwent an add-on macrolide calcineurin inhibitor tacrolimus therapy. At week 24, 40.0% of patients achieved low disease activity or remission, and the EULAR moderate or good response was 72.0% (316).

### Rituximab: Mechanism, Biomarkers and Alternative Therapy

B cells play a critical role of in pathogenesis of RA (317). In 2001, a pilot trial evaluating B cell depletive therapy in RA patients was successfully performed (318). Rituximab, which was approved by the FDA in 2006, is a chimeric mouse/human monoclonal antibody that targets the transmembrane protein CD20 molecule on the surfaces of B cells, causing apoptosis through antibody- and complement-dependent cytotoxicity (319). Rituximab monotherapy and/or in combination with methotrexate is recommended as a treatment option for RA patients who have inadequate response to TNF inhibitors and thus serves as a second-line bDMARD (320, 321). Although no fetus damage has been reported in pregnancies exposed to rituximab within 6 months, it should be considered only when no other therapeutic option is available (322). Only approximately 50% of patients achieved ACR20 response after rituximab treatment (323).

Normal cells are resistant to the complement-mediated lysis through complement regulatory proteins (CRPs), including CD55, CD59, CD46 and CD35 (324). A study showed that increased CD46 expression in peripheral B cells, but not CD35, seemed to be able to predict nonresponders (278). CD46 reduced complement-mediated lysis, one of the mechanisms of action of rituximab, thus decreasing the effectiveness of rituximab (278). It is possible that CD46 inhibitor monotherapy or combined with rituximab could be an alternative strategy for nonresponders to rituximab. Another study demonstrated that depleting CD46 from the cell surface by Ad35K++ sensitized complement-dependent cytotoxicity triggered by rituximab in CD20-positive B-cell malignancies (325, 326). However, effects of CD46 inhibition have not been validated in RA patients who do not respond to rituximab. Roles of CD55 and CD59 were also investigated in RA patients. There was no correlation between the expression levels of CD55 or CD59 at baseline or after treatment and the frequencies of B cell subsets after rituximab treatment or the extent of B cell depletion (280). Apart from these studies, persistence of switched memory B cells in lymphoid tissues was related to rituximab nonresponse (280). Structurally like infliximab, about 11% of rituximab-treated patients developed ADAs, which might influence treatment efficacy and tolerability of rituximab (282). Low or absence of baseline IFN type I response genes expression was associated with good response to rituximab (277). Decreased in expression of mammalian target of rapamycin (mTOR), p21, caspase 3, unc-51 like autophagy activating kinase 1 (ULK1), TNFα, IL-1β, and cathepsin K was predictive of better rituximab response (279). A significant reduction in circulating CD4+ T cell number was observed in RA patients with good response to rituximab (281). Depletion of CD19+/-CD27++CD38++ preplasma cells could be a predictor of good response (283).

Polymorphisms related to rituximab therapy have been well studied. The *IL-6* rs1800795-CC served as a predictor of nonresponse to rituximab in RA patients, while patients with -GC or -GG was more susceptible to rituximab (284). There was a significant correlation between this homozygosis polymorphism in the promoter region with a higher IL-6 expression level (327). It was rational that IL-6R inhibitor tocilizumab could be used as a companion to rituximab treatment in nonresponders (293, 328). *FCGR2A* polymorphism rs1801274-TT was associated with better response to rituximab (286). Several studies suggested that *FCGR3A* rs396991 genotypes, either in heterozygous or homozygous conditions, were associated with different response rates to rituximab. *FCGR3A* rs396991 G allele were associated with better response to rituximab (286, 288, 289). Paradoxically, patients with rs396991 -GT showed the highest response rate, when compared to patients with rs396991-TT or rs396991-GG (291). Homozygous carriers of the *B-cell activating factor belonging to the TNF family (BAFF)* rs9514828 C served as a better response marker to rituximab as well as the homozygotes *BAFF* rs9514828 T (292). *IRF5* rs2004640-TG (294), *TGFβ1* rs1800471-GC or -CT (295) was related to good response to rituximab. The TTTT haplotype in promoter region of B cell stimulator gene was associated with good response to rituximab therapy in RF and/or ACPA seropositive RA patients (296).

A series of studies focused on the relationships between rituximab efficacy and B cells. Incomplete depletion of baseline peripheral blood B cell receptor repertoire might predict clinical nonresponse (285). The fast rebuilding of functional B cells might be present in rituximab nonresponders (287). Total lymphocyte counts >2910/μL combined with plasmablast frequency >2.85% at baseline predicted rituximab nonresponse (290). Higher initial depth of B cell depletion was associated with good response to rituximab (297). Patients with RA who did not respond to an initial cycle of rituximab had larger circulating preplasma cell counts and incomplete B cell depletion, whereas an extra cycle of rituximab delivered prior to total B cell repopulation improved B cell depletion and clinical response (20). However, another study found that despite adequate B cell depletion, failed rituximab therapy still existed in some RA patients, and nonresponse to rituximab was associated with persistently high serum IL-6 level (293). Further, in a single-center, prospective, observational database, 51 RA patients who had discontinued rituximab therapy owing to inefficacy received either a T cell costimulation inhibitor abatacept or IL-6R inhibitor tocilizumab. After 6-month treatment, reduction of disease activity score (DAS28-ESR) and swollen joint count was more significant in tocilizumab-treated patients than in abatacept-treated patients, suggesting that IL-6-directed therapy might be a more logical and effective treatment choice than T cell costimulation blockade in RA patients with failed rituximab therapy (293). Consistently, in an investigator-led, industry-supported, prospective, longitudinal, multinational CERERRA database, 265 RA patients were analyzed and majority of them (78%) had stopped rituximab owing to ineffectiveness (328). 90 patients were prescribed abatacept, 86 were started on tocilizumab and the remaining 89 patients received TNF inhibitors (including etanercept, adalimumab, infliximab, certolizumab pegol and golimumab). After 6-month treatment, patients treated with tocilizumab had a greater decline of DAS28-ESR and better EULAR response than patients treated with TNF inhibitors or abatacept (328) (**Table 3**).

### Tocilizumab: Mechanism, Biomarkers and Alternative Therapy

IL-6 is one of the most abundant pro-inflammatory cytokines in RA. It can signal through two distinct mechanisms. In the cis-signaling, IL-6 binds to its membrane IL-6R which is mainly expressed in hepatocytes and hematopoietic cells (T cells, monocytes/macrophages, B cells and neutrophils). In the trans-signaling, IL-6 binds to its soluble IL-6R. The complex consisting of IL-6 and membrane and soluble IL-6R associates with gp130, resulting in the activation of downstream signaling events *via* JAK/STAT (329). The option to target IL-6R rather than IL-6 itself was chosen after considering that receptor concentrations have less interpatient variability than IL-6 concentrations, potentially simplifying dose and regimen selection (330). Approved by FDA in 2010, tocilizumab is the first anti-IL-6Rα humanized IgG_1_/kappa monoclonal antibody, used for the treatment of moderate to severe RA (331). Tocilizumab targets and neutralizes both soluble and membrane-bound IL-6R, resulting in inhibition of IL-6-mediated inflammatory activities (332). Tocilizumab can be either applied in combination with methotrexate or used as a monotherapy (330, 333, 334). Compared with TNF inhibitors, tocilizumab monotherapy improves the healing of focal bone erosions in RA patients and outperforms methotrexate or other csDMARDs in terms of lowering RA symptoms (330). Tocilizumab treated patients achieved an approximately 50% ACR20 response rate (335, 336).

Tocilizumab-subcutaneous and -intravenous treatment had a low immunogenicity risk, whether used alone or in combination with csDMARDs (337). The development of ADAs in a small fraction of patients had no noticeable impact on the efficacy of tocilizumab (337). Several studies investigated whether polymorphisms of genes were associated with response to tocilizumab therapy. A study with 79 RA patients enrolled reported that the major allele (A) of rs12083537 and the minor allele (C) of rs4329505 with *IL-6R* were associated with poor tocilizumab response (299). But another study with 171 RA patients enrolled found that 12083537-AA could predict better EULAR response to tocilizumab (298). Further, a larger cohort of 927 patients demonstrated no association between them (338). A genome-wide association analysis implicated the involvement of 8 loci (*CD69* rs11052877; *GalNAc-T-Like Protein 4 (GALNTL4)* rs4910008; *Ecto-NOX Disulfide-Thiol Exchanger 1 (ENOX1)* rs9594987, rs10108210 and rs703297; *Potassium Voltage-Gated Channel Interacting Protein 1 (KCNIP1)* rs703505; *C-Type Lectin Domain Family 2 Member D (CLEC2D)* rs1560011; *Solute Carrier Family 9 Member A7 (SLC9A7)* rs7055107) with response to tocilizumab (339). Relationship between *CD69* rs11052877 A alleles and good tocilizumab response was further validated in a study with 79 RA patients enrolled (300). In contrast, another study concluded that *CD69* rs11052877 A/G genetic polymorphism was not useful as a predictor of tocilizumab response in RA patients (340). Data from 87 patients suggested that *FCGR3A* rs396991-TT could be used to predict better EULAR response (286). However, no relationship between rs396991 and EULAR response was shown in a research enrolling 171 RA patients (298). Due to the small sample sizes and/or conflicting findings, larger studies are necessary to resolve whether the above genetic variations had real impact on therapeutic response to tocilizumab.

Features of pre-treatment disease activity had been demonstrated to be associated with response to tocilizumab in RA patients. A meta-analysis found that RF positivity at baseline predicted better response to tocilizumab (256). Several studies, however, found no link between RF positive and response (341, 342). A high baseline CRP level could serve as a predictor of better response to tocilizumab (302). Patients with a strong acute phase response, extra-articular symptoms, and a history of DMARDs and biological treatments may be more likely to respond quickly to tocilizumab. However, no parameter was likely to predict reaction if examined separately (343). A significantly higher proportion of patients in soluble IL-6R-low group achieved SDAI remission compared with those in soluble IL-6R-high group (303). Upregulation of gene *Interferon Alpha Inducible Protein 6 (IFI6)*, *MX Dynamin Like GTPase 2 (MX2)*, *2’-5’-Oligoadenylate Synthetase Like (OASL)* and one encoding metallothionein-1G in peripheral blood mononuclear cells was observed in tocilizumab good/moderate responders (304). Low blood D-dimer and IL-1 levels at 4 weeks were found to predict favorable treatment response to tocilizumab at 52 weeks in a population of 65 patients (305). In patients treated with tocilizumab, pre-treatment blood 14-3-3 levels predicted 1-year DAS28 remission (306). Patients with increased T Cell Receptor Alpha Variable 8-3 (TRAV8-3), EPH Receptor A4 (EPHA4), Coiled-Coil Domain Containing 32 (CCDC32), and a decrease of DHFR presented good response to tocilizumab (307). High soluble gp130Fc strongly predicted good response to tocilizumab (308). Low IL-17A level was linked to higher response rate of tocilizumab (309). A higher baseline NK cell count was associated with better clinical remission after treatment with tocilizumab (310). A serological cytokine signature showed that patients with high sICAM1 and low CXCL13 at the synovial level was negatively associated with the ACR50 response to tocilizumab, whereas patients with low sICAM1 and high CXCL13 showed good tocilizumab response (165). The presence of more TNF-induced gene transcripts in synovial samples was linked to a poor response to tocilizumab (301) (**Table 3**).

In a multicenter, retrospective study, 145 RA patients initially treated with tocilizumab (most of them discontinued tocilizumab due to lack of effectiveness) were switched to TNF inhibitors (adalimumab, certolizumab pegol, etanercept, golimumab, infliximab), abatacept or JAK inhibitors. After 24 months, drug retention was estimated. Switching to abatacept in tocilizumab-treated patients led to higher retention (315). In an open-label, non-randomized phase 3 study, 519 RA patients with inadequate response to conventional synthetic DMARDs received tocilizumab. 213 partial responders continued tocilizumab treatment and 27 nonresponders were switched to an CD20 inhibitor rituximab. At week 32, half of early partial responders benefitted from continuing tocilizumab and switching non-responders to rituximab seemed feasible (344). In a retrospective, observational clinical study, 63 nonresponders from 527 RA patients treated with tocilizumab were switched to TNF inhibitors (infliximab, adalimumab, etanercept, golimumab, and certolizumab pegol) or abatacept. The proportion of patients achieving CDAI ≤ 10 at week 24 was significantly higher in patient treated with TNF inhibitors than those treated with abatacept, and the values of the CDAI at week 24 showed the same tendency (345).

### Sarilumab: Mechanism, Biomarkers and Alternative Therapy

Sarilumab, a completely human IgG1 monoclonal antibody authorized by the FDA in 2017, specifically binds to soluble and membrane-bound IL-6R and blocks IL-6-mediated cis- and trans-signalling (346). Sarilumab presented good therapeutic efficacy when administered in combination with csDMARD in patients with inadequate response to methotrexate or at least one TNF inhibitor (346). It should be noted that sarilumab was developed in mice engineered to produce human antibodies with an affinity for human IL-6R 20-fold greater than tocilizumab (347). Preclinical findings showed that sarilumab inhibited IL-6 signaling in a dose-dependent manner at a lower concentration than tocilizumab, with no evidence of complement-dependent or antibody-dependent cytotoxicity (348). Sarilumab on the background of methotrexate significantly suppresses CRP level (349), biomarkers of bone resorption (RANKL and RANKL/OPG), bone and cartilage destruction and synovial inflammation (350). Approximately 60% of sarilumab-treated patients achieved an ACR20 response (351, 352).

Currently, only a few studies have been undertaken to identify potential biomarkers that can predict sarilumab response or nonresponse in RA patients, and alternative therapy in sarilumab nonresponders is not reported as sarilumab is approved very recently. In biomarker analysis of two phase III trials (MOBILITY involving RA patients with inadequate response to prior methotrexate and TARGET involving RA patients with inadequate response to prior TNF inhibitors), ADAs response rates were 5.6% (150 sarilumab) and 4.0% (200 mg sarilumab) and neutralizing antibodies were detected at 1.6% and 1.0% (346). Likewise, 7% of RA patients received sarilumab monotherapy in a MONARCH study exhibited an ADAs presentation but no detectable neutralizing antibodies (353). The development of ADAs was not connected with adverse effects or loss of efficacy, although it may have an impact on sarilumab pharmacokinetics (346). A phase III multicenter, randomized, controlled studies indicated that sarilumab might be more effective in RA patients who were ACPA positive (311). Also, better clinical response to sarilumab was consistently observed among patients who were RF positive and/or CCP positive in MOBILITY and TARGET studies (312). Regardless of autoantibody status, there was a more robust response in patients received 200 mg sarilumab every 2 weeks (312). Patients with elevated baseline IL-6 levels were found to have a better response to sarilumab compared to methotrexate or adalimumab than patients with normal IL-6 levels (313). Lower level of sICAM-1 at baseline was predictive of improved response to sarilumab (314) (**Table 3**).

## tsDMARDs Targeting JAKs

### Tofacitinib: Mechanism, Biomarkers and Alternative Therapy

Even through development of bDMARDs revolutionizes treatment of RA (103), these bDMARDs bring up new issues, e.g., formation of neutralizing antibodies, biologics-related toxicity and infusion-related adverse effects (354). With discovery more than 20 years ago, JAKs attract much attention since they are the most important signaling transducers (355). When triggered by cytokines such as TNF-α and IL-6, JAKs phosphorylate STATs, causing dimerization and translocation of STATs to the nucleus, where they control inflammation-related genes (7, 356). In other words, JAK inhibition blocks the action of all dependent cytokines (“many birds with one shot”) (357). The ACR and EULAR affirm that JAK inhibitors could be a viable option for RA patients who are refractory to methotrexate monotherapy and viewed on equal footing with TNF inhibitors and non-TNF biologics such as abatacept, tocilizumab and rituximab (13, 240). As the first JAK inhibitor approved by FDA in 2012, tofacitinib preferentially inhibits JAK1 and/or JAK3, and to a lesser extent of JAK2 (358, 359). Tofacitinib reduced JAK1/JAK3-mediated signaling of IL-2, IL-4, IL-6, IL-7, IL-15 and IL-21, as well as IFNα and IFNγ, resulting in the regulation of inflammatory response (360). Tofacitinib also reduced levels of CRP, C-C motif chemokine ligand (CCL)2, CXCL10, CXCL13, MMP-1 and MMP-3 (361). In the background methotrexate, either 5 mg or 10 mg tofacitinib achieved more than 50% ACR20 response in patients with methotrexate inadequate response (359).

High baseline musculoskeletal ultrasound (MSUS) and the multi-biomarker disease activity (MBDA) score could predict tofacitinib nonresponse at week 12 (362). miR-432-5p was significantly downregulated in RA patients who were responsive to tofacitinib therapy (363). Lower levels of IFN-γ, IL-6, IL-17 and higher levels of IL-35 were found in tofacitinib responders than in nonresponders (364). Lower age, CRP, ACPA, and DKK-1 indicated the good treatment effects of tofacitinib therapy on bone mineral density changes (365). MMP-3 had higher pre-treatment values correlating with improved tofacitinib response (361). Clinical improvement with tofacitinib therapy correlated with reductions in STAT1 and STAT3 phosphorylation (361). The available evidence is insufficient to support alternative therapy for tofacitinib because limited clinical trials have been conducted (13) (**Table 4**).

**Table 4:** Potential biomarkers for response or partial response/nonresponse to tsDMARDs targeting JAKs.

tsDMARDs	Biomarkers for response	Sample size	Reference	Biomarkers for partial response/nonresponse	Sample size	Reference
Tofacitinib	Downregulated miR-432-5p	16	(363)	High baseline MSUS and MBDA score	25	(362)
	Lower levels of IFN-γ, IL-6, IL-17 and higher levels of IL-35	32	(364)			
	Lower age, CRP, ACPA, and DKK-1	26	(365)			
	Higher pre-treatment MMP-3 values	14	(361)			
	Reductions in STAT1 and STAT3 phosphorylation	14	(361)			
Baricitinib	High titers of CarbV IgA and IgG antibodies	584	(366)	Previous use of bDMARDs (non-TNF inhibitors) or JAK inhibitors	113	(367)
	No previous targeted DMARD (b or tsDMARDs) use	113	(367)			
	Lower DAS28-CRP score at baseline	113	(367)			
Upadacitinib	Higher levels of IL-17A, IL-17C, CCL11, CCL20, and TIMP4	300	(368)	No data	N/A	N/A

ACPA, Anti–citrullinated protein antibody; CCL, C-C Motif Chemokine Ligand; CRP, C-reactive protein; DKK, Dickkopf; IFN, interferon; JAK, Janus kinase; MBDA, Multi-Biomarker Disease Activity; MMP, matrix metalloproteinase; MSUS, Musculoskeletal Ultrasound; STAT1, signal transducer and activator of transcription 1; TNF, tumour necrosis factor.

### Baricitinib: Mechanism, Biomarkers and Alternative Therapy

Approved by FDA in 2018, baricitinib is the second JAK inhibitor for RA treatment that selectively and reversibly inhibits JAK1 and JAK2 and then modulates JAK-STATs intracellular signaling pathways (369, 370). Baricitinib also inhibits the effects of JAK3, TyK2, tyrosine-protein kinase Met (c-MET) and Checkpoint kinase 2 (Chk2) (371). Baricitinib could reduce pannus formation and bone damage in multiple murine models of arthritis (371), and also present an osteoprotective effect, increasing mineralization in bone-forming cells in phase III studies (372). After treatment, mean serum values of IgG, IgM and IgA were decreased and remained stable below baseline (373). Treatment with baricitinib did not result in increased autoantibody (RF and ACPA) titers (374). Caution is recommended when leflunomide or teriflunomide was co-administered with baricitinib (373). Baricitinib demonstrated a consistent, beneficial treatment effects in bDMARD-refractory patients (375). Baricitinib also ameliorated disease progression in RA patients who were naïve to DMARDs or respond inadequately to csDMARDs, and the beneficial effects were similar to those observed with adalimumab (376). In two clinical trials, RA patients treated with baricitinib achieved ACR20 for 77% and 55%, respectively (377, 378).

High titers of anti-carbamylated vimentin (CarbV) IgA and IgG antibodies were associated with a greater clinical response to baricitinib as measured by SDAI and DAS28-high-sensitivity CRP (366). Patients who had previously used bDMARDs (non-TNF inhibitors) or JAK inhibitors had decreased rates of DAS28-CRP improvement when treated with baricitinib (367). No previous targeted DMARD (b or tsDMARDs) use was associated with the achievement of low disease activity (367). However, this result was contradicted to another study in which baricitinib nonresponse was not related to prior use of one or more of bDMARDs (375). Besides, baseline characteristics (excluding DAS28-CRP score) did not substantially affect the clinical response to baricitinib in RA patients (379), but a lower DAS28-CRP score at baseline was associated with the achievement of low disease activity (367). Taken together, predicting response to baricitinib by previous treatment with DMARDs and baseline characteristics still needs further investigation (**Table 4**).

### Upadacitinib: Mechanism, Biomarkers and Alternative Therapy

Unlike tofacitinib (359) and baricitinib (380), upadacitinib is engineered to be selective for JAK1 and serves as the second-generation JAK inhibitor approved by FDA in 2019 for RA (381). The rationale for selectively targeting JAK1 is that the anti-inflammatory effect should be maintained *via* inhibiting JAK1, but effects on undesired JAK2- and JAK3-dependent processes should be minimized (382, 383). Upadacitinib was shown to be >40-fold, 130-fold and 190-fold more selective for JAK1 versus JAK2, JAK3 and TYK2, respectively (383). Upadacitinib may be used as monotherapy or in combination with methotrexate in active RA patients with inadequate response to cs or bDMARDs (384). It has been demonstrated that upadacitinib was more effective than adalimumab in RA patients who were concomitantly receiving methotrexate (221). Upadacitinib could decrease circulating numbers of lymphocytes, neutrophils and NK cells with no significant changes in RF and ACPA levels (383, 385–387). In three studies (SELECT-NEXT, SELECT-BEYOND and SELECT-MONOTHERAPY), rates of ACR20 were 64%, 65% and 68% in RA patients treated with 15 mg upadacitinib, and 66%, 56% and 71% in patients treated with 30 mg upadacitinib, respectively (388–390).

Clinical response with upadacitinib treatment was mainly associated with slightly higher levels of the IL-17A, IL-17C, CCL11, CCL20, and Tissue inhibitor of metalloproteinases-4 (TIMP4) (368). A study showed that adalimumab appeared to affect M1 macrophages, while upadacitinib appeared to affect T cells preferentially (368), which was in line with another study (391). This modulatory pattern by upadacitinib was consistent with its wide cytokine receptor inhibition profile as compared to specific TNF inhibition and could account, at least in part, for superior efficacy of upadacitinib over adalimumab (368). In a randomized, double-blinded SELECT-COMPARE study enrolled 651 upadacitinib-treated patients, a total of 39% (252/651, including non- and incomplete-responders) patients were randomized to adalimumab. Low disease activity was achieved in 36% nonresponders and 45% incomplete-responders after switching for 6 months (392) (**Table 4**).

## Precision Medicine and RA

Over the past decades, treatment of RA always depends on “trial-and-error” methods of finding a DMARD that works (393). Regarding the above-mentioned clinical trials enrolling RA patients who have failed initial DMARDs therapy, we can see that successive conventional or biologic switching, either within the same or different mechanistic class, is advocated as an alternative therapy by an experience-oriented principle. This leads to the apparent disadvantage of continuously exposing patients to multiple drugs that they do not respond to, with unnecessary side-effects, delaying the use of effective agents, and causing a serious economic burden to society (19). According to the current ACR and EULAR guidelines for managing RA, it is still a great challenge to choose the right treatment for the right patients.

**Figure 1:**
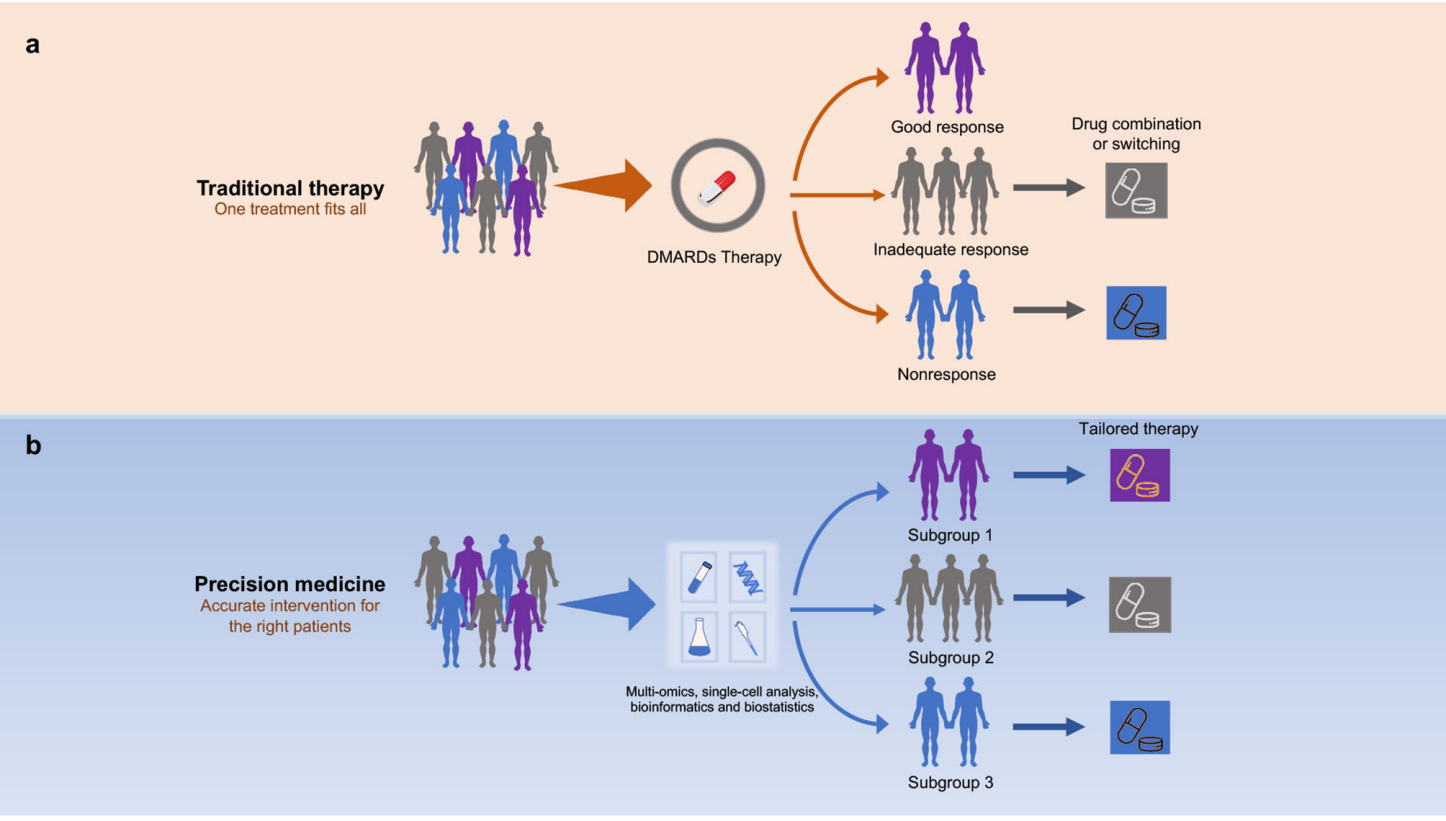
Comparison between traditional therapy and precision medicine.

The most recent EULAR recommendations provide an updated research agenda that highlights important issues to be addressed in the future, such as the safety and efficacy of various drug sequences and combinations, the discovery of biomarkers to stratify patients and predict therapeutic response, and the reasons for efficacy loss (13). It is worth noting that these issues fall into the category of the emerging precision medicine approach for disease treatment, which is a medical model that proposes the customization of healthcare, with diagnosis, prognosis and treatment being tailored to different subgroups of patients (394). Precision medicine in RA has been recently discussed regarding its great potential in allowing a better diagnosis (RA vs. non-RA), finding biomarkers for preferential treatment selection in patients (responders vs. nonresponders), as well as understanding the prognosis of the disease (progressor vs. non-progressor) (24). Precision medicine is believed to lead to the next revolution for overcoming treatment failure in RA, with the introduction of cutting-edge technologies and big data, especially the multi-omics, single-cell analysis, bioinformatics and biostatistics (**Figure 1**). 

## Multi-Omics in Precision Medicine of RA

Multi-omics is the integration of datasets generated from genomics, transcriptomics, epigenomics, proteomics and metabolomics (395). There has been a growing trend of studies, which utilize high-throughput multi-omics analyses to achieve personalized health care, especially through prediction of disease risk and early intervention for a potentially better outcome (396). Despite much hoopla based primarily on oncology data, the progress of multi-omics in autoimmunity is currently restricted (397). Previously, genomics, transcriptomics and epigenetics have been used separately to characterize the molecular basis of treatment efficacy in RA patients (145, 274, 398–400). However, the molecular effects of DMARDs from multi-omics perspectives are unknown. Recently, researchers reported longitudinal monitoring of the drug response at multi-omics levels in RA patients (401). They revealed that DMARDs treatments altered the molecular profile of RA patients closer to that of healthy individuals at the transcriptome, serum proteome, and immunophenotype levels. Effects of infliximab and tocilizumab on this molecular profile which defined stable clinical remission were greater than that of methotrexate. Tocilizumab normalized some specific molecular markers that methotrexate and infliximab did not modify, implying that tocilizumab was a more potent treatment for RA at the molecular level (401). Moreover, researchers also identified molecular signatures in transcripts and serum proteins that were resistant to DMARDs. These signals were linked to RA independently of known disease severity indices and were mostly explained by a neutrophil, monocyte, and lymphocyte imbalance. This knowledge will facilitate the identification of biomarkers and drug discovery and contribute to the development of precision therapy for RA (401). Besides, another study performed a sequential multi-omics analysis integrating transcriptomics and genomics to identify gene signatures associated with the response to anti-TNF therapy in RA patients (402). Using transcriptomic data from the RA synovium, thirteen gene co-expressed modules were found to be associated with anti-TNF response. At the genetic level, two of these modules were confirmed to be associated with the anti-TNF response using two independent GWAS cohorts of RA patients. Functional analysis suggested that nucleotide metabolism and Tregs could mediate the response to anti-TNF therapy. These findings demonstrated the existence of a drug-specific genetic foundation for an anti-TNF response, allowing for therapy stratification in the quest for response biomarkers in RA (402). In addition, the latest study conducted multi-omics and machine learning to predict response to anti-TNF therapies in RA patients. Transcription and/or DNA methylome signatures were found to be associated with response to different anti-TNF therapy in peripheral blood mononuclear cells (PBMCs), monocytes, and CD4+ T cells from RA patients (403). Further, transcription signatures in responders to adalimumab and etanercept were divergent in PBMCs, and this phenomenon was reproduced in monocytes and CD4+ T cells. Differentially methylated sites in etanercept responders but not in adalimumab responders were substantially hypermethylated (403). Finally, machine learning models based on these molecular signatures were built to reliably predict response prior to anti-TNF treatment, paving the way for tailored anti-TNF therapeutic treatment regimens (403).

## Single-Cell Analysis in Precision Medicine of RA

Unlike traditional omics research, researchers have discovered that cells differ dramatically at the transcriptome, proteomic, and epigenomic levels among tissues, organs, organ systems, and organisms. Individual immune cell coordination is crucial in RA for the production of efficient immune responses to infections while immunological tolerance is maintained to protect the host. Furthermore, when immune responses are dysregulated, pathologically essential cells may constitute only a minor proportion of the immune system. Examining the roles of particular immune cells in etiology, disease progression, and medication failure should yield valuable insights into RA (404). Single-cell analysis investigates genomes, transcriptomics, proteomics, metabolomics, and cell-cell interactions in individual cells, leading to a higher resolution of cellular distinctions and a better understanding of an individual cell activity in the context of its microenvironment (405–407). It enables the high-dimensional dissection of single cells at multi-omics levels, which could facilitate the discovery of new biomarkers and stratified RA patients into more precise subgroups (408). As mentioned in a subsection of certolizumab pegol, the circulating T1IFN ratio linked to remarkably diverse gene expression patterns in monocytes of RA patients, and certain transcripts such as JAK1 were very informative and could indicate alternative treatment paths in individuals anticipated to be non-responders to anti-TNF therapy. This work was done mainly using a novel single-cell PCR approach, which was similar to single-cell sequencing (175). Another study described a robust statistical method to test for disease associations with single-cell data called MASC (Mixed-effects modeling of Associations of Single Cells). This approach enabled modeling of technical and inter-individual variance as random effects, allowing robust detection of disease-associated cellular populations. Using MASC to analyze CD4+ T cells from blood of RA patients, researchers discovered a population of memory CD4+ T cells known as CD27-HLA-DR+ that was enlarged in the circulation of RA patients. Further, CD27-HLA-DR+ T cells were enriched within inflamed RA joints, rapidly produced IFN-γ and cytolytic factors, and contracted with successful treatment of RA (409). Furthermore, the repertoires of B cell receptors, which may be collected using single cell-resolution sequencing technology, carry a personal history of antigen exposure for a donor (410). Single-cell sequencing of plasmablasts derived from RA patients revealed the presence of B cell receptors specific for CCP and other RA-associated autoantigens (411). Plasmablasts from ACPA-positive patients were sequenced, and both IgA-secreting and IgG-secreting clones were found to be responsive to common RA autoantigens (412). A longitudinal analysis of plasmablasts from individuals with clinically apparent RA revealed the presence of persistent IgA-producing cells that underwent ongoing affinity maturation and produced ACPAs (413). As the complex relationship between the response of DMARDs and the ACPA/anti-CCP has been discovered, single-cell functional B cell receptors sequencing is likely to provide new insights into precision medicine of RA.

## Bioinformatics and Biostatistics in Precision Medicine of RA

Nowadays, there is an exponentially increasing number of databases integrating personal omics and volume of healthcare data (414, 415). However, due to the nature and complexity of such data, immediate interpretation or usage by healthcare practitioners is frequently out of the question. Biostatistics and bioinformatics pertain to the acquisition and interpretation of the quantitative data. No sharp delineation exists between them. Bioinformatics tends to deal with data in many dimensions, so-called “big data” (416), while biostatistics is a building block for the complex data analytics methods in bioinformatics (417). Integration of bioinformatics and biostatistics facilitate the establishment of sophisticated methods based on omics and advanced mathematics, such as artificial intelligence, machine learning and deep learning (415, 418–420). In a nutshell, artificial intelligence seeks to increase cognitive abilities and performance of computers in order to tackle complicated and massive data-oriented challenges by identifying interaction patterns among variables, learning from experiences, planning, and anticipating better directions. Machine learning is a subfield of artificial intelligence that employs and proposes various algorithms for learning from large amounts of data and revealing multifaceted relationships between data features in order to predict accuracy in various contexts and support decision-making processes, whereas deep learning is a dominant approach based on artificial neural networks (421). These methods have been widely utilized in oncology field to identify individuals at risk, to predict which prevention strategies work best on patients, to automatically screen different subtypes of diseases, or to perform drug repurposing (422). In recent years, application of these methods in RA is burgeoning. A study, for example, offered an automatic method for detecting RA disease activity in an electronic medical record. Different machine learning models were developed and tested using a training set of clinical notes and laboratory data. The models extracted terms such as synovitis, pain, or stiffness as input features by using a text analysis tool on clinical notes, and also used laboratory values of CRP or ESR. Disease activity of each RA patient was predicted by different DAS28 score. This study demonstrated that automatically discovering RA disease activity from electronic medical record data was, in principle, a learnable task, with results approximating human performance (423). Deep learning was also applied to forecast RA disease activity. Researchers classified disease activity into two categories: remission/low and moderate/high. Demographics, past CDAI score, ESR and CRP level, DMARDs, oral and injectable glucocorticoids, and autoantibodies (RF and/or ACPAs) were all taken into account. Results showed that CDAI was the most important feature for prediction of disease, followed by cortisone injections and CRP (424). Deep learning algorithms have been utilized in image processing to find patterns in images (so-called convolutional neural networks). This sort of neural network has been utilized to detect bone erosions (425) and differentiate RA patients from healthy participants from conventional hand radiographs (426). As discussed in the above subsection of multi-omics in precision medicine of RA, the combination of multi-omics and/or clinical data with machine learning could be used to predict response to DMARDs in RA patients (403). Transcription and/or DNA methylome signatures were found to be associated with response to different anti-TNF therapy in PBMCs, monocytes, and CD4+ T cells from RA patients (403). Machine learning models based on these molecular signatures were developed to accurately predict response before anti-TNF treatment. In another study, researchers used a regression model to predict the response to anti-TNF therapy after methotrexate failure, considering of demographic and clinical data in addition to genetic data (single nucleotide polymorphisms). The model classified the response to anti-TNF treatment with 78% accuracy (427).

## Conclusion and Perspectives

Over the past few decades, researchers have delivered continuous efforts as above mentioned toward overcoming treatment failure in RA. In fact, these efforts have driven the diagnosis, treatment and prognosis of RA entering an early stage of precision medicine, which was commonly described as personalized medicine prior to the proposal of precision medicine. These efforts lead to a massive step when compared to the earlier era in which RA is seen as a devastating and stubborn disease. While gratifying, researchers have realized that even through sequential development of csDMARDs, bDMARDs and tsDMARDs has gradually improved treatment outcome of RA, response rates seem to reach ceiling (approximately 40-60%) in different clinical trials with DMARDs monotherapy or combination therapy in RA patients. Notably, this response ceiling is observed irrespective of the mode of action of the different types of DMARDs or their diverse specific cellular, molecular and signaling targets, such as CD20, TNF, IL-6, CD80-CD86, and the JAK-STAT pathways (18, 428). Although emerging data suggest that a higher response threshold could be reached, breaking through the response ceiling has been proven particularly difficult. This may be because that, on one hand, RA is a highly heterogeneous and complex disease with unclear understanding of mechanisms. On the other hand, small sample sizes and insufficient technologies lead to conflicting or uncertain conclusions as well as slow renewal of knowledge in basic research and translational studies of RA. Precision medicine refers more appropriately to the generation of criteria for advanced taxonomy of patients, producing models to identify and classify clinical decisions for each disease phenotype. This new perspective on patient evaluation can make use of both fundamental laboratory and clinical analyses as well as large data provided by cutting-edge technologies, as previously discussed. Precision medicine is exhibiting great potentials to be a more efficient way to overcome the treatment failure and has begun to emerge in RA studies. Undoubtedly, there are also some limitations of precision medicine, such as high cost of sequencing, existence of ethical issues, difficulty in collection and storage of large amount of data, and lack of easy and standardized approaches for data interpretation for doctors and other healthcare providers. It is believed that most of them will be solved with the rise of new technologies and algorithms. In the foreseeable future, RA patients will ultimately be precisely classified, receive their tailored therapy, and avoid wasting time during months or years of ineffective treatment. Precision medicine will also generate sufficient data to elucidate the molecular foundation underlying drug failure and push the development of next-generation DMARDs.

## Author Contributions

CL, AL, and DH supervise and revise the manuscript. ZW writes and edits the manuscript. JH and DX provides their professional expertise. All authors contributed to the article and approved the submitted version.

## Funding

This review is supported by the Natural Science Foundation Council of China (81700780, 81922081, 82172386, 81774114 and 82074234), The Science, Technology and Innovation Commission of Shenzhen (JCYJ20210324104201005), the Croucher Foundation (Gnt#CAS14BU/CAS14201), the 2020 Guangdong Provincial Science and Technology Innovation Strategy Special Fund (Guangdong-Hong Kong-Macau Joint Lab) (2020B1212030006) and the National administration of Traditional Chinese Medicine, regional Chinese medicine (Specialist) diagnosis and Treatment Center Construction project rheumatology.

## Conflict of Interest

The authors declare that the research was conducted in the absence of any commercial or financial relationships that could be construed as a potential conflict of interest.

## Publisher’s Note

All claims expressed in this article are solely those of the authors and do not necessarily represent those of their affiliated organizations, or those of the publisher, the editors and the reviewers. Any product that may be evaluated in this article, or claim that may be made by its manufacturer, is not guaranteed or endorsed by the publisher.
